# Effect of yeast species and processing on intestinal microbiota of Atlantic salmon (*Salmo salar*) fed soybean meal-based diets in seawater

**DOI:** 10.1186/s42523-023-00242-y

**Published:** 2023-04-04

**Authors:** Jeleel O. Agboola, Sérgio D. C. Rocha, Dominic D. Mensah, Jon Ø. Hansen, Ove Øyås, David Lapeña, Liv T. Mydland, Magnus Ø. Arntzen, Svein J. Horn, Margareth Øverland

**Affiliations:** 1 Faculty of Biosciences, Department of Animal and Aquacultural Sciences, Norwegian University of Life Sciences, P.O. Box 5003, NO-1432 Ås, Norway; 2 Faculty of Chemistry, Biotechnology and Food Science, Norwegian University of Life Sciences, P.O. Box 5003, NO-1432 Ås, Norway

**Keywords:** *Cyberlindnera jadinii*, *Wickerhamomyces anomalus*, Gut microbiota, Predicted metabolic capacity, SBMIE, Microbial diversity, Inactivated, Autolysis

## Abstract

**Background:**

Yeasts are gaining attention as alternative ingredients in aquafeeds. However, the impact of yeast inclusion on modulation of intestinal microbiota of fish fed plant-based ingredients is limited. Thus, the present study investigates the effects of yeast and processing on composition, diversity and predicted metabolic capacity of gut microbiota of Atlantic salmon smolt fed soybean meal (SBM)-based diet. Two yeasts, *Cyberlindnera jadinii* (CJ) and *Wickerhamomyces anomalus* (WA), were produced in-house and processed by direct heat-inactivation with spray-drying (ICJ and IWA) or autolyzed at 50 °C for 16 h, followed by spray-drying (ACJ and AWA). In a 42-day feeding experiment, fish were fed one of six diets: a fishmeal (FM)-based diet, a challenging diet with 30% SBM and four other diets containing 30% SBM and 10% of each of the four yeast products (i.e., ICJ, ACJ, IWA and AWA). Microbial profiling of digesta samples was conducted using 16S rRNA gene sequencing, and the predicted metabolic capacities of gut microbiota were determined using genome-scale metabolic models.

**Results:**

The microbial composition and predicted metabolic capacity of gut microbiota differed between fish fed FM diet and those fed SBM diet. The digesta of fish fed SBM diet was dominated by members of lactic acid bacteria, which was similar to microbial composition in the digesta of fish fed the inactivated yeasts (ICJ and IWA diets). Inclusion of autolyzed yeasts (ACJ and AWA diets) reduced the richness and diversity of gut microbiota in fish. The gut microbiota of fish fed ACJ diet was dominated by the genus *Pediococcus* and showed a predicted increase in mucin O-glycan degradation compared with the other diets. The gut microbiota of fish fed AWA diet was highly dominated by the family *Bacillaceae*.

**Conclusions:**

The present study showed that dietary inclusion of FM and SBM differentially modulate the composition and predicted metabolic capacity of gut microbiota of fish. The inclusion of inactivated yeasts did not alter the modulation caused by SBM-based diet. Fish fed ACJ diet increased relative abundance of *Pediococcus,* and mucin O-glycan degradation pathway compared with the other diets.

**Supplementary Information:**

The online version contains supplementary material available at 10.1186/s42523-023-00242-y.

## Background

Plant protein sources are increasingly being used in commercial aquafeeds [1, 2]. Among the plant-based ingredients, the use of soybean meal (SBM) in diets of Atlantic salmon is restricted due to the presence of anti-nutritional factors (such as trypsin inhibitors, protease inhibitors and saponin) that compromise the growth performance, nutrient digestibility, and health of fish [3, 4]. A number of studies [5–10] have reported that dietary inclusion of SBM induce inflammation in the distal intestine of Atlantic salmon; a condition widely known as SBM-induced enteritis (SBMIE), which is characterized by loss of enterocyte vacuolization, reduction in mucosal fold height, and infiltration of inflammatory cells in the lamina propria and epithelial submucosa. Considering these limitations, a refined soy-product known as soy-protein concentrate (SPC) with low level of anti-nutritional factors, is currently used in commercial salmon diets. The use of plant ingredients such as SPC in aquafeeds also raises ethical and environmental concerns as continuous use of SPC in aquafeeds may increase pressure on cultivable land, water and energy use, as well as decrease their availability for direct human consumption [11, 12]. Therefore, there is emerging need for sustainable novel ingredients for aquaculture, which can be used to improve the utilization and/or as alternatives to conventional plant-based ingredients in fish feeds.

Microbial ingredients such as yeasts are gaining attention as potential novel ingredient in aquaculture due to their ability to convert low-value by-products into high-value resources [13], high nutritional values [14–16], low environmental footprint [17] and functional effects in fish [18, 19]. Studies have shown that dietary inclusion of yeasts could alleviate adverse effects of SBM in Atlantic salmon [18, 19], but little is known of their effects on intestinal microbiota of fish. The gut microbiota plays important roles in host physiology and metabolic processes, such as digestive function, growth performance, immune function, and health [20–22]. A number of studies [23–26] have documented the effects of SBM inclusion on intestinal microbiota of Atlantic salmon. Identifying microbiota modulated by inclusion of yeasts in the diets may be crucial for improving nutrient utilization, growth performance, and health of Atlantic salmon fed plant-based diets. Therefore, the objective of the present study was to examine the effect of yeast species and processing on richness, diversity and predicted metabolic profile of gut microbiota of Atlantic salmon fed SBM-based diet in seawater. Two yeasts, *Cyberlindnera jadinii* (CJ) and *Wickerhamomyces anomalus* (WA) produced from wood sugars using in-house bioreactors, were used in the current study.

## Methods

### Yeasts, experimental diets, and fish feeding trial

The CJ and WA yeast biomass were produced in a 30 L bioreactor using a growth medium composed of a blend of enzymatic hydrolysates of pre-treated Norwegian spruce wood (*Picea abies*) and chicken by-products as described by Lapeña et al. [13]. After harvesting, the yeasts were processed following the protocol described by Agboola et al. [18]. Briefly, the yeast biomass was washed, centrifuged and the resulting paste was divided into two equal parts. One part of the yeast paste was directly inactivated with a spray-dryer (SPX 150 MS, SPX Flow Technology, Denmark) set at 180 °C and 80 °C for inlet and outlet temperature, respectively. The other half of the yeast paste was autolyzed at 50 °C for 16 h in a stirred 30 L reactor (Einar, Belach Bioteknik, Sweden), followed by spray-drying using the same conditions as above. The resulting processed yeast products were: inactivated CJ (ICJ), autolyzed CJ (ACJ), inactivated WA (IWA), and autolyzed WA (AWA). The nutritional and cell wall compositions of the four yeast products are presented in Additional file 1: Table S1.

Six experimental diets were formulated to meet or exceed [27, 28] the nutritional requirements of Atlantic salmon smolts; a fishmeal-based (FM) control diet, a challenging diet containing 30% soybean meal (SBM) and four diets containing 30% SBM with 10% inclusion of the different processed yeasts (ICJ, ACJ, IWA and AWA). Table 1 shows the ingredient and analyzed compositions of the six experimental diets. The diets were cold-pelleted using a P35A pasta extruder (Italgi, Carasco, Italy) and dried at 60 °C in small experimental driers until the pellets reached a moisture content of less than 10%. The production of the experimental diets is fully described in Agboola et al. [29].

**Table 1:** Diet formulation and nutritional composition of the experimental diets*

	FM	ICJ	ACJ	IWA	AWA	SBM
*Diet formulation (g/kg)*						
Fish meal^a^	433.4	208.4	208.4	208.4	208.4	261.4
Soybean meal^b^	0	300	300	300	300	300
Wheat gluten meal^c^	170	111	111	111	111	136
Potato starch^d^	120	68	68	68	68	90
Cellulose	80	0	0	0	0	0
Yeast^m^	0	100	100	100	100	0
Fish oil^e^	130	130	130	130	130	130
Gelatin^f^	60	60	60	60	60	60
Monocalcium phosphate^g^	0	10	10	10	10	10
Premix^h^	5	5	5	5	5	5
L-lysine^i^	0	3	3	3	3	3
DL-Methionine^j^	0	3	3	3	3	3
Choline chloride^k^	1.5	1.5	1.5	1.5	1.5	1.5
Yttrium^l^	0.1	0.1	0.1	0.1	0.1	0.1
*Analyzed diet composition (g/kg dry matter (DM) unless otherwise stated)*						
Dry matter (g/kg)	926.6	889.9	889.2	924.5	913.9	897.3
Crude protein	531.8	518.3	530.3	519.5	521.4	542.6
Starch	131.9	92.6	93.3	89.3	87.6	103.6
Ash	78.3	74.7	74.8	73.7	73.5	77.2
Carbon	509.1	502.5	517.8	513.1	511.0	509.7
Sulphur	6.0	6.2	6.0	6.1	6.0	6.3
Energy (MJ/kg DM)	23.3	23.3	23.3	23.1	23.1	23.1
DP:DE^o^	23.1	22.8	22.8	22.5	22.5	23.3

^a^LT fishmeal, Norsildmel, Egersund, Norway; ^b^Soybean meal, Denofa AS, Fredrikstad, Norway; ^c^Wheat gluten, Amilina AB, Panevezys, Lithuania; ^d^Lygel F 60, Lyckeby Culinar, Fjälkinge, Sweden; ^e^NorSalmOil, Norsildmel, Egersund, Norway; ^f^Rousselot 250 PS, Rousselot SAS, Courbevoie, France; ^g^Monocalcium phosphate, Bolifor MCP-F, Oslo, Norway Yara; ^h^Premix fish, Norsk Mineralnæring AS, Hønefoss, Norway. Per kg feed; Retinol 3150.0 IU, Cholecalciferol 1890.0 IU, α-tocopherol SD 250 mg, Menadione 12.6 mg, Thiamin 18.9 mg, Riboflavin 31.5 mg, d-Ca-Pantothenate 37.8 mg, Niacin 94.5 mg, Biotin 0.315 mg, Cyanocobalamin 0.025 mg, Folic acid 6.3 mg, Pyridoxine 37.8 mg, Ascorbate monophosphate 157.5 g, Cu: CuSulfate 5H_2_O 6.3 mg, Zn: ZnSulfate 151.2 mg, Mn: Mn(II)Sulfate 18.9 mg, I: K-Iodide 3.78 mg, Ca 1.4 g; ^i^L-Lysine CJ Biotech CO., Shenyang, China; ^j^Rhodimet NP99, Adisseo ASA, Antony, France; ^k^Choline chloride, 70% Vegetable, Indukern SA., Spain; ^l^Y_2_O_3_. Metal Rare Earth Limited, Shenzhen, China

^m^ICJ—inactivated *Cyberlindnera jadinii*; ACJ—autolyzed *C. jadinii*; IWA—inactivated *Wickerhamomyces anomalus*; AWA—autolyzed *W. anomalus*

^o^DP:DE = Digestible protein to digestible energy ratio. Calculated using internal digestibility values of various ingredients

^*^The diets are: FM—fishmeal-based; SBM—soybean meal-based; 4 other diets containing 300 g/kg SBM and 100 g/kg of ICJ, ACJ, IWA and AWA yeasts

A 42-day seawater feeding trial with Atlantic salmon smolts (initial body weight = 136 ± 0.25 g) was conducted at the research facility of the Norwegian Institute of Water Resources (NIVA, Solbergstrand, Norway). A total of 450 vaccinated salmon smolts were randomly allocated into 18 fiber tanks (300 L) and fed one of the six experimental diets (n = 3 tanks per diet) for 6 h per day using automatic feeders delivering feed every 12 min. The fish were reared under a 24 h light regime in a flow-through system with an average water temperature of 11.5 °C and average oxygen saturation of 84%. The water flow was kept at an average of 5.5 L min^−1^ during the experimental period. Water salinity was gradually increased from 5 ppt at the start, until it reached full salinity (33 ppt) during the first 12 days of the experiment.

### Sample collection

Six fish were randomly selected from each tank, anaesthetized with metacaine (MS-222, 50 mg L^−1^ water), and killed with a sharp blow to the head for digesta sampling. After dissection, the distal intestine was opened longitudinally and the digesta was carefully removed using sterile plastic spatulas. The digesta was placed in cryotubes, snap-frozen in liquid nitrogen and stored at -80 °C. To obtain sterile conditions, tools were cleaned and decontaminated using 70% ethanol and flaming between each fish. Additionally, feed and water samples were collected into sterile plastic containers and stored at -80 °C. Water samples were collected from both the source tank and the fish rearing tanks.

### DNA extraction

Total DNA was extracted from ~ 200 mg of digesta (18 samples per dietary group) and 100 mg of ground feed (3 replicates per diet) using QIAamp® Fast DNA Stool Mini Kit (Qiagen, Hilden, Germany, Cat. No. 51604) following the manufacturer’s specifications with some modifications as described elsewhere [30]. In addition to the digesta and feed samples, total DNA was extracted from the water samples. 500 mL each of source water (2 samples) and rearing tank water (4 samples) were filtered through a MF-Millipore membrane filter with 0.22 µm pore size (Sigma-Aldrich, Cat. No GSWP04700) and total DNA was extracted using the same protocol described above. The rearing water (500 mL from each tank) samples were mixed, and four sub-samples (500 mL each) were taken and used for the DNA extraction. Total DNA was also extracted from blank filter paper used for the filtration of water samples. For quality control of the present workflow, a microbial community standard (mock), which consists of eight bacteria and two yeasts (ZymoBIOMICS™, Zymo Research, California, USA; Cat. No. D6300) was included for DNA extraction as positive control. In addition, a blank negative control was added to each batch of DNA extraction by omitting the input material. Total DNA were extracted from blank control, mock positive control and blank filter paper following the method used for digesta, feed and water samples. The DNA concentration of all the samples were measured in duplicates using Invitrogen™ Quant-iT™ Qubit™ dsDNA HS (High Sensitivity) assay kit (Thermo Fisher Scientific, California, USA, Cat. No. Q32854) with the Qubit 4 Fluorometer (Invitrogen™). The extracted DNA were stored at -20 °C until further analysis.

### PCR amplification

The V3-V4 hypervariable regions of the bacterial 16S rRNA gene were amplified in a 25 µL reaction volume containing 2 × KAPA HiFi HotStart Ready Mix (12.5 µL) (Roche Sequencing Solutions, Mat. No. 7958935001), DNA template (5 µL), and 1.33 µM primers (3.75 µL of each primer). The primers used for the amplicon PCR were 341F (5’-CCT ACG GGN GGC WGC AG-3’) and 785R (5’-GAC TAC HVG GGT ATC TAA TCC-3’). The amplification was set at initial denaturation of 95 °C for 3 min; 25 cycles of denaturation at 95 °C for 30 s; annealing at 55 °C for 30 s; extension at 72 °C for 30 s; followed by a final extension at 72 °C for 5 min. After the amplification process, duplicate PCR products were pooled and purified using Agentcourt AMPure XP beads (Beckman Coulter, Indiana, USA, Cat. No. A63881), and the cleaned PCR products were examined by 1% agarose gel electrophoresis.

### Library preparation and sequencing

The sequencing was carried out on a Miseq platform following the Illumina 16S metagenomic sequencing library preparation protocol [31]. The cleaned PCR amplicons were multiplexed by dual indexing using the Nextera Index Kit v2 Set A (Illumina, California, USA, Cat. No. FC-131-2001). The index PCR products were cleaned using the AMPure beads and quantified using the Invitrogen™ Quant-iT™ Qubit™ dsDNA BR (Broad range) assay kit (Thermo Fisher Scientific, California, USA, Cat. No. Q32853) with the Qubit 4 Fluorometer (Invitrogen™). To determine the library size representative, cleaned libraries were selected and analyzed using the Agilent DNA 1000 Kit (Agilent Technologies, California, USA, Cat. No. 067-1505). The libraries were diluted to 4 nM in 10 mM Tris (pH 8.5) and pooled in an equal volume. The blank control samples with library concentrations lower than 4 nM were pooled directly without further dilution. The pooled library was denatured using 0.2 N NaOH. Due to low diversity of the amplicon library, 5% Illumina generated PhiX control (Illumina, San Diego, Waltham, MA, USA, Cat No: FC-110-3001) was spiked in by combining 570 μL amplicon library with 30 μL PhiX control. The library was then loaded at 8 pM and sequenced on the Miseq System (Illumina, San Diego, California, USA) using the Miseq Reagent Kit v3 (600-cycle) (Illumina; catalog no., MS-102–3003). The sequencing was done in two runs. To prevent potential batch effects between sequencing runs, the digesta and the feed samples were distributed between the runs with consideration that each dietary treatment and each experimental tank were equally represented. Also, water and control samples were evenly distributed between the two runs.

### Sequence data processing

The sequence data were processed in R (version 4.0.5) [32]. For each sequencing run, DADA2 was used to process the raw sequence data and generate amplicon sequence variants (ASVs) [33]. Briefly, the demultiplexed pair-ended reads were trimmed off the primer sequences (first 17 bps of forward reads and first 21 bps of reverse reads), truncated at the position where the median Phred quality score crashed (forward reads at position 300 bp and reverse reads at position 230 bp for both runs) and filtered off low quality reads. After the trimming and filtering, a model of error rates was developed to remove error sequences. The forward and reverse reads were merged, and the ASV table for each run was constructed. The ASV table for each sequencing run were merged, and assigned with taxonomy using the reference database, SILVA version 138.1 [34, 35]. A phyloseq object was constructed from the generated ASV table, the taxonomy table and the sample metadata using the phyloseq R package (version 1.34.0) [36]. Taxa identified as chloroplasts or mitochondria were removed from the ASV table. The ASVs that had no phylum-level taxonomic assignments or appeared in less than three biological samples were conservatively filtered from the ASV table. The contaminating ASVs due to reagent contamination and cross contamination were identified and removed from ASV table as described elsewhere [37]. The ASVs were then clustered using VSEARCH algorithm and subsequently curated with LULU [38]. The post-clustering ASV table and representative sequences were used for the downstream data analysis. The core ASVs and alpha-diversity indices (observed ASVs, Pielou’s evenness, Shannon’s index and Faith’s phylogenetic diversity (PD)) were computed according to Li et al. [37]. Similarly, the beta-diversity indices (Jaccard distance, unweighted UniFrac distance, Aitchison distance and PhILR transformed Euclidean distance) were computed following Li et al. [37]. The Jaccard distance and unweighted UniFrac distance were calculated by rarefying the ASV table into minimum sequence size i.e., 1,604 reads per sample (Additional file 1: Fig. S1). Conversely, Aitchison distance and PhILR transformed Euclidean distance were computed using the unrarefied ASV table.

### Metabolic reaction analysis of gut microbiota

The metabolic reaction analysis of gut microbiota was performed according to the method described by Yilmaz et al. [39]. The ASVs for the digesta samples were mapped to metabolic reactions using an available collection of genome-scale metabolic models (GSMMs) of gut microbes [40]. Only ASVs that could be mapped to family or lower taxonomic rank and to at least one GSMM were included in the reaction level analysis. For each sample, we calculated the normalized abundance of each reaction *i*:

$${a}_{r}\left(i\right)=\frac{\sum_{j=1}^{n}{a}_{\mathrm{ASV}}\left(j\right)E(i,j)}{\sum_{j=1}^{n}{a}_{\mathrm{ASV}}\left(j\right)}$$

where ${a}_{\mathrm{ASV}}(j)$ is the abundance of ASV *j* in the sample, *n* is the total number of ASVs, and $E(i,j)$ is the expected probability (frequency of occurrences) of reaction *i* in the GSMMs mapped to ASV *j*.

### Statistical analysis

The statistical difference among the dietary groups for the microbial compositions at genus or lowest taxonomy ranks (top 15 most abundant taxa) were evaluated using Kruskal–Wallis test, followed by multiple comparison using Wilcox pair-wise comparison test. Similarly, the alpha-diversity measurements were evaluated using Kruskal–Wallis test and statistical differences among the dietary groups were detected using Wilcox pair-wise comparison test. The statistical difference among the dietary groups for the beta-diversity indices were computed using permutation multivariate analysis of variance (PERMANOVA) [41] with 999 permutations using the R package vegan 2.5.7 [42], followed by a pair-wise comparison. Principal coordinates analysis (PCoA) was used to visualize the beta-diversity indices. The homogeneity of multivariate dispersions among the dietary groups was computed by permutation test, PERMDISP [43], using the R package vegan [42] and visually assessed with boxplots. Significant differences with adjusted *p* < 0.05 among dietary groups were detected using the Benjamini–Hochberg procedure [44]. For the metabolic reaction analysis, mean abundance of each reaction was tested using a two-sample *t*-test for each pair of diets. Multiple testing was corrected using the Benjamini–Hochberg procedure [44] and reactions with adjusted *p* ≤ 0.05 were considered to be significantly different between diets. For each pair of diets, the enriched pathways among the significantly different reactions were computed using Fisher’s exact test. The pathways with adjusted *p* ≤ 0.05 based on Benjamini–Hochberg procedure were considered to be enriched. Additionally, principal component analysis (PCA) was performed separately on standardized ASVs (Additional file 1: Fig. S2) and reaction abundances (z-scores) (Additional file 1: Fig. S3).

## Results

To aid the readers understanding of the data reported in this study, results on growth performance, nutrient digestibility, intestinal histopathology, immunological and transcriptomic changes of fish fed the experimental diets have been reported with more detail elsewhere [29]. In brief, fish grew from 136 ± 0.25 g initial average weight to 179 ± 7.06 g average final weight after 42 days of feeding the experimental diets. The inclusion of yeasts did not compromise growth performance of fish. Histological and immunohistochemistry examination showed that the inclusion of CJ yeast reduced loss of supranuclear vacuolization and decreased population of CD8α positive cells in the distal intestine of fish fed SBM-based diets. Inclusion of both yeasts (CJ and WA) induced transcriptomic changes associated with wound healing and immune response pathways in fish fed SBM-based diets.

### Characteristics of sequence data

After the sequence denoising, ASV filtering and clustering, a total number of 6.6 million reads were retained for the downstream data analysis. The median of reads per sample used for downstream analysis was 23,087, with the minimum and maximum values being 1,604 and 180,844, respectively. The reads for the downstream analysis generated a total of 906 unique ASVs, of which 76.4% were assigned at the genus level and 13.5% annotated at the species level.

### Microbiota composition of mock and negative controls

All the eight bacterial species expected in the mock were successfully identified at genus level, with only *Staphylococcus aureus* being identified at species level (Additional file 1: Fig. S4). The relative abundance of *S. aureus* was correctly estimated, whereas the abundance of *Salmonella*, *Pseudomonas* and *Escherichia-Shigella* were overestimated. Contrary, the relative abundance of *Listeria*, *Lactobacillus*, *Enterococcus* and *Bacillus* were underestimated. The average Pearson correlation coefficient (Pearson’s *r*) between the expected and the observed taxonomic profile of the mock was 0.30, whereas the Pearson’s *r* between the observed mock was 0.99. The dominant taxa identified as contaminants in the negative controls and the blank filter papers were *Actinobacteria* (47%), *Bacilli* (18%), and *Gammaproteobacteria* (15%) (Additional file 2: Table S2).

### Microbiota associated with feed and water

**Fig. 1:**
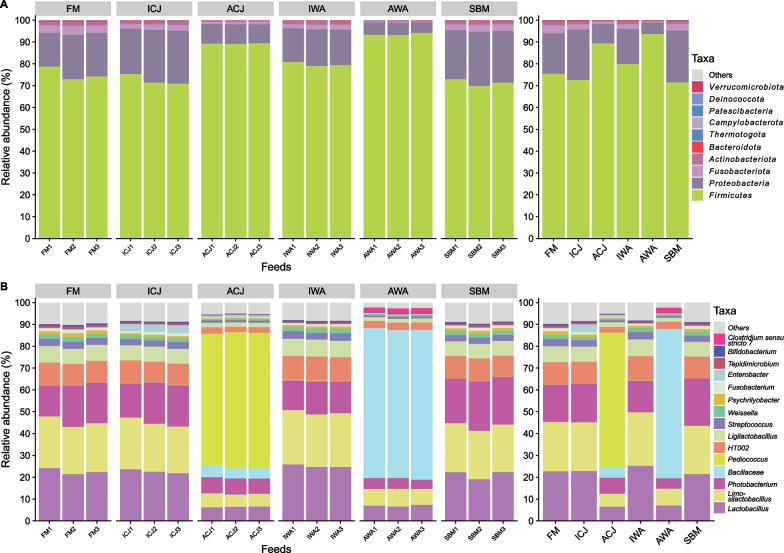
Microbiota composition in the feed samples. Relative abundance of the top 10 most abundant taxa at phylum level (**A**) and top 15 most abundant taxa at genus or lowest taxonomic rank (**B**). The mean relative abundance of each taxon within the same diet is displayed on the right side. The samples are grouped by diets; FM—fishmeal-based; SBM—soybean meal-based; 4 experimental diets containing 300 g/kg SBM and 100 g/kg of ICJ—inactivated *Cyberlindnera jadinii*; ACJ—utolyzed *C. jadinii*; IWA—inactivated *Wickerhamomyces anomalus*; AWA—autolyzed *W. anomalus* diets

At phylum level, the feed-associated microbiota was dominated by *Firmicutes* and *Proteobacteria* (Fig. 1A). The ACJ (89%) and AWA (94%) feeds had higher abundance of *Firmicutes* compared with the remaining feeds (72–80%). The relative abundance of *Proteobacteria* was lower in ACJ (9%) and AWA (5.3%) feeds compared with the remaining diets (16–24%) (Fig. 1A). At genus or lowest taxonomic rank, the ACJ and AWA feeds were dominated by *Pediococcus* (62%) and *Bacillaceae* (68%), respectively (Fig. 1B). The microbiota composition in FM, ICJ, IWA and SBM feeds were dominated by *Lactobacillus* (21–25%), *Limosilactobacillus* (22–25%), *Photobacterium* (15–22%), *HT002* (10–11%) and *Ligilactobacillus* (6.7–7.7%) (Fig. 1B).

The microbiota in the source water was dominated by phyla *Proteobacteria* (55%), *Actinobacteriota* (14%) and *SAR324 clade (Marine group B)* (14%), whereas the taxonomic compositions of the rearing tank water were dominated by phyla *Proteobacteria* (55%) and *Bacteroidota* (31%) (Additional file 1: Fig. S5A). At the genus or lowest taxonomy level, *SUP05 cluster* (13%), *Candidatus Actinomarina* (10%) and *Clade II* (9%) dominated the microbiota in the source water (Additional file 1: Fig. S5B). The microbiota in the rearing tank water were dominated by the taxa *Sulfitobacter* (11%), *Colwellia* (7%), *Hellea* (7%), *Lacinutrix* (5%) and *Maribacter* (5%) (Additional file 1: Fig. S5B). *Bacillaceae* (0.01–0.2%) and *Pediococcus* (0.02–2%) were detected in both source water and tank water.

### Digesta-associated microbiota

**Fig. 2:**
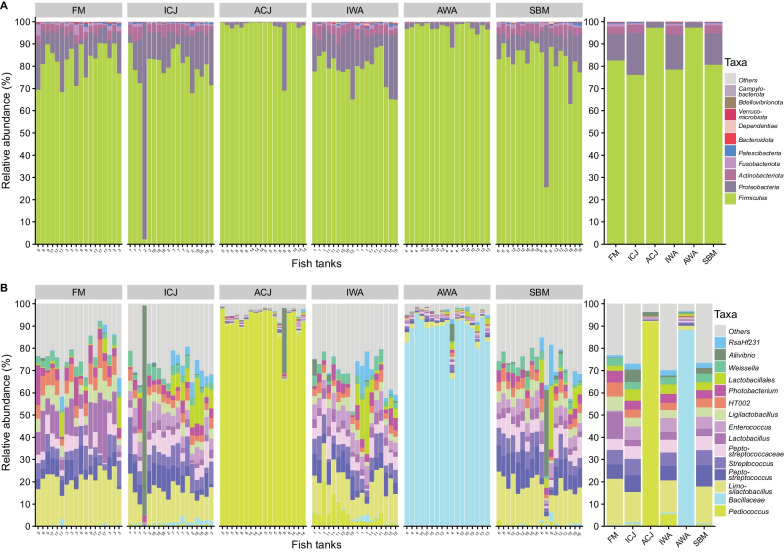
Microbiota composition in the digesta of fish fed the experimental diets. Relative abundance of the top 10 most abundant taxa at phylum level (**A**) and top 15 most abundant taxa at genus or lowest taxonomic rank (**B**). The mean relative abundance of each taxon within the same diet is displayed on the right side. The samples are grouped by diets; FM—fishmeal-based; SBM—soybean meal-based; 4 experimental diets containing 300 g/kg SBM and 100 g/kg of ICJ—inactivated *Cyberlindnera jadinii*; ACJ—autolyzed *C. jadinii*; IWA—inactivated *Wickerhamomyces anomalus*; AWA—autolyzed *W. anomalus* diets

Regardless of the diets, the taxonomic compositions of the digesta samples at phylum level were dominated by *Firmicutes*, *Proteobacteria* and *Actinobacteriota* (Fig. 2A). Fish fed ACJ (97%), and AWA (97%) had higher abundance of *Firmicutes* compared with those fed the other diets (76–81%) (Fig. 2A). Fish fed ACJ (2.5%) and AWA (2.2%) diets had lower composition of *Proteobacteria* compared with fish fed the other diets (12–19%) (Fig. 2A). *Actinobacteriota* composition in the digesta of fish fed ACJ (0.2%) and AWA (0.4%) diets was lower compared with fish fed the remaining diets (3.3–4.1%) (Fig. 2A).

**Fig. 3:**
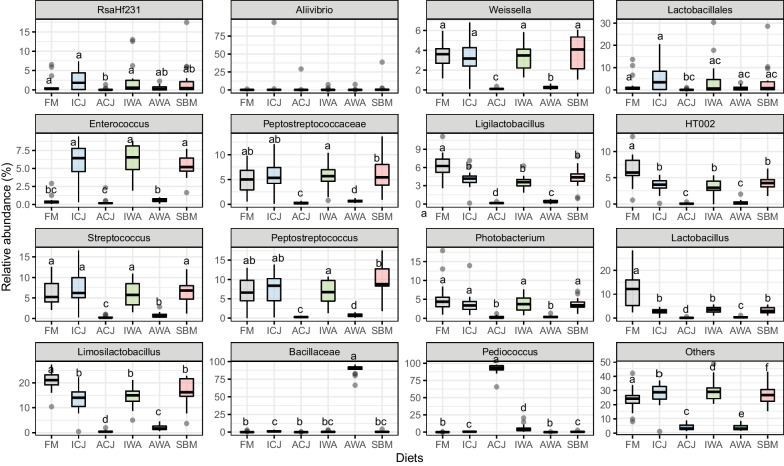
Boxplots of relative abundance of the top 15 most abundant taxa (at genus or lowest taxonomic rank) in the digesta of fish fed the experimental diets. The samples are grouped by diets; FM—fishmeal-based; SBM—soybean meal-based; 4 experimental diets containing 300 g/kg SBM and 100 g/kg of ICJ—inactivated *Cyberlindnera jadinii*; ACJ—autolyzed *C. jadinii*; IWA—inactivated *Wickerhamomyces anomalus*; AWA—autolyzed *W. anomalus* diets. Different lower-case letters represent taxa with significantly different (*p* < 0.05) relative abundance among the diets

The taxonomic composition of digesta samples at the genus or lowest taxonomy rank was influenced by the dietary group (Figs. 2B & 3). Fish fed ACJ (92%) diet were significantly dominated by *Pediococcus* compared with the other diets (Figs. 2B & 3). Similarly, fish fed AWA (88%) diet were significantly dominated by *Bacillaceae* compared with fish fed the other diets (Figs. 2B & 3). *Lactobacillus* (12%) and *Limosilactobacillus* (21%) were significantly higher in fish fed FM compared with fish fed the other diets (Figs. 2B & 3). Fish fed ICJ, IWA and SBM diets (5.4–6.3%) had significantly higher abundant of *Enterococcus* compared with the other diets (Figs. 2B & 3). *Streptococcus*, *Peptostreptococcus*, *HT002*, *RsaHf231*, *Weissella* and *Photobacterium* were significantly higher in fish fed FM, ICJ, IWA and SBM diets compared with fish fed ACJ and AWA diets (Figs. 2B & 3).

**Fig. 4:**
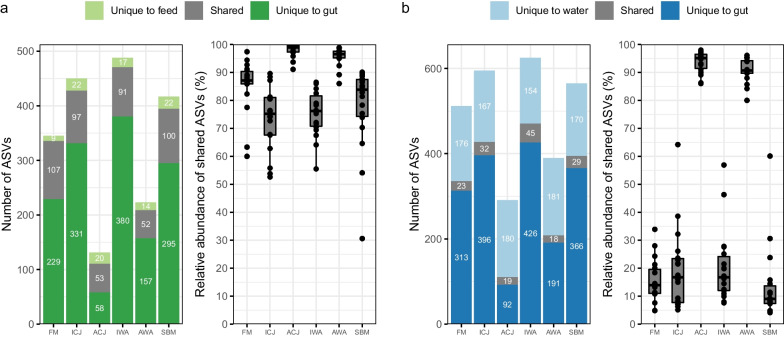
The microbial overlap between the gut and feeds (**a**) and between the gut and the water (**b**). The number of shared amplicon sequence variants (ASVs) is shown in the left figure of each panel. The relative abundance of shared ASVs is shown in the right figure of each panel. The minimum relative abundance of ASVs to be considered as present in a sample was 0.05%. The samples are grouped by diets; FM—fishmeal-based; SBM—soybean meal-based; 4 experimental diets containing 300 g/kg SBM and 100 g/kg of ICJ—inactivated *Cyberlindnera jadinii*; ACJ—autolyzed *C. jadinii*; IWA—inactivated *Wickerhamomyces anomalus*; AWA—autolyzed *W. anomalus* diets

When comparing the ASVs of the gut, water and feed, the composition of the gut microbiota was similar to that of the feed, but different from the water microbiota (Fig. 4). The ASVs overlap between the gut and the feed was higher than between the gut and water.

### Core microbiota

In total, 94 ASVs were identified as core microbiota (present in 80% of the digesta samples) in fish fed the experimental diets (Additional file 1: Fig. S6A-B; Table S3). Fifteen ASVs classified as *Peptostreptococcus*, *Limosilactobacillus*, *Weissella*, *Ligilactobacillus*, *Streptococcus* and *Lachnospiraceae* were identified to be present in all the dietary groups. Fish fed FM and SBM diets shared 37 primary core ASVs, belonging to members of *Peptostreptococcus*, *Photobacterium*, *RsaHf231*, and lactic acid bacteria (LAB) including *Streptococcus*, *Lactobacillus*, *Limosilactobacillus*, *Weissella*, *Ligilactobacillus* and *HT002*.

### Alpha-diversity

**Fig. 5:**
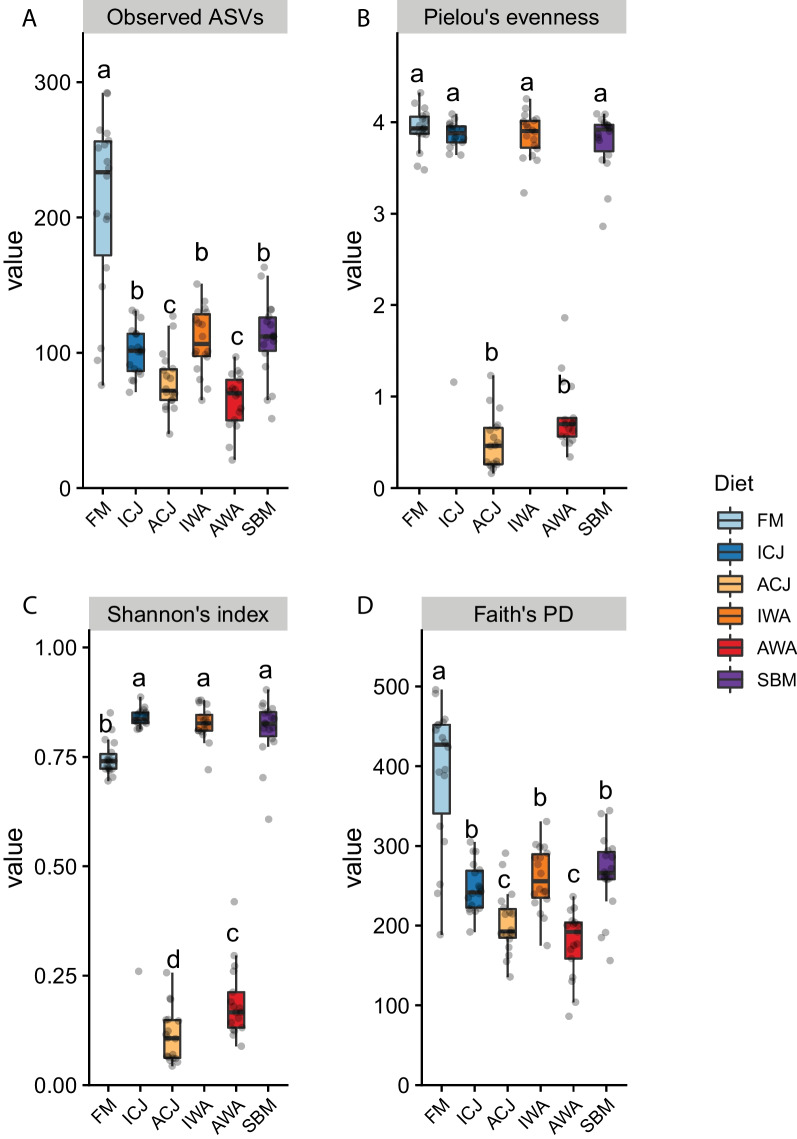
Boxplots of alpha-diversity of gut microbiota of fish fed the experimental diets. The four alpha-diversity indices used are; (**a**) observed amplicon sequence variants (ASVs), (**b**) Pielou’s evenness (**c**) Shannon’s index and (**d**) Faith’s phylogenetic diversity (PD). The samples are grouped by diets; FM—fishmeal-based; SBM—soybean meal-based; 4 experimental diets containing 300 g/kg SBM and 100 g/kg of ICJ—inactivated *Cyberlindnera jadinii*; ACJ—autolyzed *C. jadinii*; IWA—inactivated *Wickerhamomyces anomalus*; AWA—autolyzed *W. anomalus* diets. Indices with different lower-case letters are significantly different (*p* < 0.05) among the diets

Based on the four indices, the microbial diversity of fish fed ACJ and AWA diets was significantly lower compared with fish fed the other diets (Fig. 5; Additional file 1: Table S4). The observed ASVs and Faith’s PD showed that fish fed FM diet had significantly higher microbial diversity compared with fish fed ICJ, IWA and SBM diets (Fig. 5A, D). Contrarily, based on Shannon’s index, the microbial diversity of fish fed FM diet was significantly lower compared with those fed ICJ, IWA and SBM diets (Fig. 5C). Excluding fish fed ACJ and AWA diets, the microbial diversity was similar among the other diets based on Pielou’s evenness (Fig. 5B). The microbial compositions of fish fed ICJ, IWA and SBM were similar based on the four alpha-diversity indices (Fig. 5).

### Beta-diversity

**Fig. 6:**
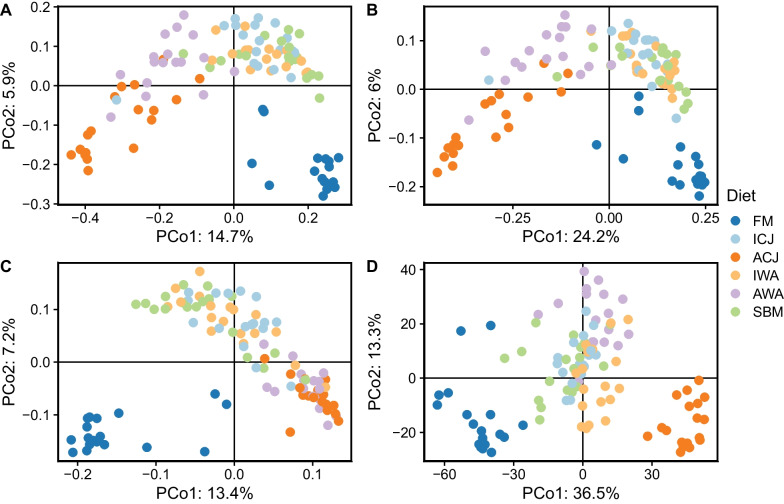
Principal coordinates (PCo) analysis plots of beta-diversity of gut microbiota of fish fed the experimental diets. The four beta-diversity indices used are; (**A**) Jaccard distance, (**B**) Unweighted Unifrac distance (**C**) Aitchison distance and (**D**) PhILR transformed Euclidean distance. The samples are grouped by diets; FM—fishmeal-based; SBM—soybean meal-based; 4 experimental diets containing 300 g/kg SBM and 100 g/kg of ICJ—inactivated *Cyberlindnera jadinii*; ACJ—autolyzed *C. jadinii*; IWA—inactivated *Wickerhamomyces anomalus*; AWA—autolyzed *W. anomalus* diets

The PCoA plots built on the four beta-diversity indices showed that the microbiota of fish fed FM diet were clearly distinct from the other diets (Fig. 6). Based on the four beta-diversity indices, the PCoA plots showed that microbiota of fish fed ICJ, IWA and SBM diets were similar, and clearly clustered from those fed FM, ACJ and AWA diets (Fig. 6A-D). The PCoA plots based on Jaccard distance, unweighted UniFrac distance and PhILR transformed Euclidean distances showed separation of microbiota in fish fed ACJ diet compared with fish fed AWA diet (Fig. 6A, B, D). On the contrary, the microbiota of fish fed ACJ diet were similar compared with fish fed AWA diet based on Aitchison distance matrix (Fig. 6C). The PERMANOVA tests showed that beta-diversity were significantly influenced by the dietary groups, and the results were in line with the PCoA plots (Additional file 1: Table S5). Based on the four distance matrices, the microbiota of fish fed FM diet were significantly different from those fed the other diets. Also, the PERMANOVA tests showed similarity in the microbiota of fish fed ICJ, IWA and SBM diets, which were different from those fed ACJ and AWA diets. The statistical tests showed that the microbiota of fish fed ACJ diet were significantly different from fish fed AWA diet. The tests for homogeneity and multivariate dispersions are presented in Additional file 1: Fig. S7 and Table S6. The multivariate dispersions were significantly affected by the dietary groups based on the four distance matrices.

### Metabolic capacity of gut microbiota

Fifty-eight percent (526) of the 906 ASVs identified in the current study could be mapped to at least one model from a published collection of GSMMs of gut microbiota. Thirty-seven percent (338), 19% (176) and 1.3% (12) of the ASVs were matched to family, genus, and species, respectively (Additional file 1: Fig. S8A). The ASVs matched to family, genus, and species were mapped to an average of 16, 13 and 1 model(s), respectively (Fig. S8B). The models mapped to ASVs contained 4802 different reactions, half of which (55%) were present in all samples. Most samples (90%) contained more than 90% of the reactions, but the abundances of many reactions differed significantly between samples and diets. Furthermore, the variability in the data could be explained in a few components using PCA of reaction abundances rather than ASV abundances (Additional file 1: Figs. S2 and S3).

**Fig. 7:**
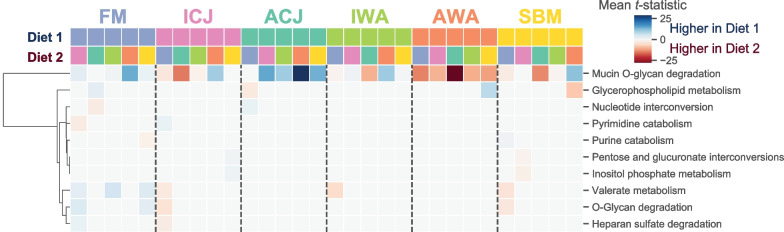
Hierarchical clustering of the significantly enriched metabolic subsystems between each pair of dietary groups. Columns are diet pairs, rows are metabolic subsystem, and the color of each cell indicates whether the metabolic subsystem was enriched in diet 1 (blue) or diet 2 (red). FM—fishmeal-based; SBM—soybean meal-based; 4 experimental diets containing 300 g/kg SBM and 100 g/kg of ICJ—inactivated *Cyberlindnera jadinii*; ACJ—autolyzed *C. jadinii*; IWA—inactivated *Wickerhamomyces anomalus*; AWA—autolyzed *W. anomalus* diets

By classifying the reactions into metabolic pathways, ten pathways were enriched in pairwise comparisons between the dietary groups (Fig. 7). The differences in mean abundance of enriched pathways for each pair of diets are presented in Additional file 1: Fig. S9. The gut microbiota of fish fed FM diet showed predicted enrichment of metabolic pathways related to mucin O-glycan degradation, valerate metabolism and O-Glycan degradation, as well as lower enrichment of purine and pyrimidine catabolism pathways compared with fish fed ICJ and SBM diets (Fig. 7 & Additional file 1: Fig. S9A, E). The gut microbiota of fish fed ACJ diets showed predicted enrichment of mucin O-glycan degradation pathway compared with fish fed ICJ, IWA, AWA and SBM diets (Fig. 7 & Additional file 1: Fig. S9). The predicted enrichment of metabolic pathways was similar for fish fed FM and ACJ diets, except for glycerophospholipid pathway (enriched in fish fed FM) and nucleotide interconversion (enriched in fish fed ACJ) (Fig. 7 and Additional file 1: Fig. S9B).

## Discussion

### Core microbiota

In line with previous studies [45–47], *Limosilactobacillus*, *Weissella*, *Ligilactobacillus* and *Streptococcus* were annotated as core microbiota in the present study and are commonly identified in the intestine of Atlantic salmon reared in seawater [23, 37, 45]. These taxa belong to the group of lactic acid bacteria (LAB), which are known to promote beneficial health effects in fish [48–50]. It is important to note that prior to seawater transfer, fish were reared under the same environmental conditions and fed a fishmeal/fish oil-based commercial diet in freshwater. Therefore, the presence of LAB as the core microbiota after the feeding experiment could be attributed to the composition of the commercial diet (particularly lipid content) and the environmental conditions (e.g., temperature, dissolved oxygen, pH etc.) used during the pre-experimental stage. *Peptostreptococcus* and *Lachnospiraceae* were also identified as core taxa in the present study. These taxa have been found in the intestinal digesta of Atlantic salmon but are rarely identified as core microbiota [37, 45, 46]. *Lachnospiraceae* are associated with production of short chain fatty acids (butyrate) [51], and has been reported to play a role in preventing inflammatory diseases in fish [52]. It is noteworthy to state that, *Mycoplasma* which is commonly reported as core microbiota in the intestine of both wild and farmed Atlantic salmon [37, 47, 53–58], was not identified in the present study. It is unclear why *Mycoplasma* was not detected, but it might be linked to the differences in environmental factors during the early life stages of fish such as live food, feeds, water temperature and salinity or simply lack of exposure to *Mycoplasma*. These factors are reported to influence the establishment of core microbiota in fish [48, 56–60]. Also, a recent study has demonstrated that the establishment of *Mycoplasma* increased with time in seawater [58], implying that the experimental duration may be too short for its establishment in the gut of fish used in the current experiment.

### Soybean meal has a dominating effect on modulation of gut microbiota

In accordance with previous findings in fish [23–26, 61–63], the present study observed differences between the gut microbiota of fish fed FM diet compared with those fed SBM diet. The microbial richness and diversity were higher in fish fed FM diet compared with fish fed SBM diet, which is in line with previous studies [23, 24]. Most of the microbial taxa found in Atlantic salmon gut such as *Lactobacillus, Limosilactobacillus, Ligilactobacillus, HT002,* and *Vagococcus* were more abundant in fish fed FM diet compared with fish fed SBM diet. The current results showed that the microbiota of fish fed SBM were dominated by LAB such as *Lactobacillus, Limosilactobacillus, Ligilactobacillus, Weissella, Enterococcus* and *Streptococcus*, which is in accordance with previous findings [23–25]. The high abundance of LAB in fish fed SBM-based diet has been attributed to the presence of soluble and insoluble oligosaccharides such as raffinose and stachyose, which can be used as substrates for metabolism and growth by the microbiota [23]. Results from the present study published elsewhere [29] showed that fish fed SBM diet developed typical signs of SBMIE and as previously mentioned, LAB are generally considered as beneficial microbes promoting intestinal health and growth of fish. Although members of LAB, such as some species of *Enterococcus* and *Streptococcus,* are considered pathogenic, it seems counterintuitive that LAB enrichment could be observed in fish that developed SBMIE. This observation challenges the general understanding that microbiota play a role in the development of SBMIE in fish. The relationship between increased relative abundance of LAB and development of SBMIE has been documented in previous studies [23, 24, 26]. Reveco et al. [24] speculated that the increased relative abundance of LAB could be related to their capability to produce antimicrobial peptides (such as bacteriocins) against the certain bacteria in fish presenting SBMIE. Also, during the development of SBMIE, it is possible that the commensal bacteria (LAB) have less competition and more opportunity to proliferate. It remains unclear whether the increase in relative abundance of LAB is a cause or a consequence of the inflammatory response in fish presenting SBMIE. Further investigation is needed to clarify the role of intestinal microbiota in the development of SBMIE in Atlantic salmon fed plant-based diets.

The present study revealed that microbial richness and diversity were similar among fish fed ICJ, IWA and SBM diets. This implies that the inclusion of inactivated yeasts (CJ and WA) did not modulate the intestinal microbiota of fish fed SBM diet. This contradicts previous findings which showed that feeding diets containing *Saccharomyces cerevisiae* and WA yeasts modulated the intestinal microbiota in rainbow trout [64, 65]. It is worthy of note that SBM was not used in the previous studies [64, 65]. In line with our present results, dietary supplementation of mannan oligosaccharides (MOS) from yeasts did not modulate microbial diversity and richness of gilthead sea bream fed SBM-based diet [66]. These contradicting results underscore the importance of ingredients used in diet formulation with respect to possible effects of yeast or its cell wall components on gut microbiota of fish [67]. The cell wall polysaccharides of yeasts such as glucans and MOS can serve as substrates for microbial growth [68–70], and as a consequence modulates the intestinal microbiota in fish fed yeast-based diets [64, 65]. However, our speculation is that 30% inclusion level of SBM possibly has a dominating effect in modulating gut microbiota when compared with 10% inclusion level of inactivated yeasts in the current study. Study on the effects of inactivated yeasts (CJ and WA) in Atlantic salmon fed SBM-free diets is recommended in the future. Despite the similarity in microbial composition of fish fed ICJ, IWA and SBM diets, the results of the present study reported elsewhere [29] showed that inclusion of inactivated yeasts (CJ and WA) dampened the inflammatory response in the distal intestine of fish fed SBM diet. Therefore, it can be hypothesized that the ameliorating effects of inactivated yeasts on SBMIE is related to their capability to stimulate immune responses rather than through modulation of intestinal microbiota in Atlantic salmon.

### Autolyzed yeasts modulate gut microbiota of fish

The results of the present study revealed that the gut bacteria composition of fish fed ACJ and AWA diets were greatly affected by the diets when compared with the other groups. The ACJ and AWA diets promoted the dominance of genus *Pediococcus* and the family *Bacillaceae*, respectively. Such modulation consequently led to a decrease in richness and diversity of gut microbiota of fish fed ACJ and AWA diets compared with fish fed the remaining diets. A previous study reported that autolyzed *S. cerevisiae* reduced the microbial diversity of gilthead sea bream fed commercial-like diet [71].

The increased relative abundance of *Pediococcus* and *Bacillaceae* in Atlantic salmon fed the autolyzed yeasts may be explained by the autolytic conditions, feed-borne microbiota and/or feed composition. Based on BLAST analysis using the NCBI website, the *Pediococcus* ASV in our data set revealed sequence homologous to *Pediococcus acidilactici* and *P. claussenii*, whereas the *Bacillaceae* ASVs matched a wide range of members in the *Bacilli* microbial clade, including *Caldibacillus pasinlerensis*, *C. thermoamylovorans, Cerasibacillus terrae, C. quisquiliarum, Alkalihalobacillus gibsonii* and *A. lonarensis*. Optimum growth temperature for the genus *Pediococcus* [72] and the family *Bacillaceae* [73] ranged between 30 and 60 °C. Thus, it is plausible that the growth of spores of these microbial taxa were selectively promoted during the autolytic process (at 50 °C for 16 h). Although thermal conditions during the spray-drying were expected to inactivate the microbes in the yeast, dead or bacterial spores can still be profiled by the DNA sequencing methods. We could assert that the inclusion of autolyzed yeasts promotes the enrichment of a certain microbial taxon in the digesta of fish, but the effects seem to be yeast dependent. Therefore, the observed dominance of these microbial taxa in the gut of fish fed ACJ and AWA feeds probably reflects not only the active microbes, but also dead microbes and spores transferred from the yeasts into the feeds. In future studies, analyzing the microbes in the yeast cream and the dried yeasts would further elucidate the extent to which the diet effects are attributable to the transfer of microbes from the yeasts to the diets. Techniques such as viability PCR and RNA sequencing [74], which are able to distinguish dead or active microbes, would provide useful information regarding the role of yeast- and feed-associated microbes in shaping the intestinal microbiota of fish fed yeast-based diets. Changes in cell wall polysaccharide of autolyzed yeasts may also partly contributed to the observed dominance of *Pediococcus* and *Bacillaceae* in fish fed ACJ and AWA diets. Previous studies have reported that the solubility [75] and biophysical properties [18, 76] of cell wall polysaccharides of yeasts are modified by the autolytic process. It is possible that the glucans and MOS in autolyzed yeasts are more available as substrates for the intestinal microbiota compared with intact yeasts. In the current study, it was impossible to distinguish whether the substrates for microbiota growth and metabolism were derived from SBM or from the yeast. Thus, the extent to which the modification of cell wall polysaccharides of yeasts contributed to the intestinal microbiota of fish could not be ascertained. This hypothesis can be tested by supplementing autolyzed yeasts to SBM-free diets and sequencing the intestinal microbiota of fish fed these diets.

It remains unclear whether the high abundance of a single taxon in fish fed ACJ or AWA diet was beneficial or caused dysbiosis in the host. The species *P. acidilactici* and *Bacillus subtilis* are among the most widely studied probiotic bacteria and have been reported to promote growth performance, nutrient digestion, disease resistance and intestinal health in farmed fish [20, 77–80]. Based on this, it was expected that the high relative abundance of *Pediococcus* and *Bacillaceae* in fish fed the autolyzed yeasts would enhance the performance and intestinal health compared with fish fed the other diets. This was not the case, based on the results of fish performance and intestinal health presented in Agboola et al. [29]. Fish performance was unaffected by the dietary treatments, and the inclusion of autolyzed yeasts in fish fed SBM did not alleviate SBMIE beyond the level observed for fish fed SBM with inactivated yeasts [29]. Therefore, it is possible that the physiological response of fish to high relative abundance of both *Pediococcus* and *Bacillaceae* is limited by low feed intake [29] and short experimental period used in the current study. Long-term experiments with *ad-libitum* fish feeding of diets containing autolyzed yeasts is recommended in the future. Also, it could simply be that the microbiota are dead and without probiotic effects in fish. The lack of difference in physiology of fish fed inactivated and autolyzed yeasts also supports the hypothesis that the dominance of a single taxon in the gut of fish fed ACJ and AWA is due to transfer of bacteria spores from the feeds to the fish gut. Thus, the reproducibility of microbiota modulated in fish fed yeast-based diets in the present study should be investigated in future studies. It is important to note that no mortality or noticeable signs of disease were recorded, suggesting that the high abundance of a single taxon in fish fed ACJ and AWA diets in the present study did not lead to dysbiosis.

### Gut microbiota is driven by feed microbiota and less by water microbiota

Feed and rearing water are two environmental factors shaping the intestinal microbiota of fish [81–90]. In agreement with previous studies in fish [81–86], there was high overlap between microbiota in the gut and the feeds. Still, it is unclear to what extent the carry-over microbes from the feeds influenced the intestinal microbiota. It would be interesting in the future to stain for live/dead gut bacteria and then use fluorescence-activated cell sorting followed by 16S rRNA sequencing to identify the dead spores from the live bacteria. In the current study, microbial overlap between the intestine and the feeds was higher than the microbial overlap reported elsewhere [30, 86] in Atlantic salmon fed insect-based diets. The discrepancy can be attributed to the feed processing technology used in these studies. Contrary to the present study, feeds used in the previous studies [30, 86] were processed using extrusion technology. Extrusion is a hydrothermal process that is capable of inactivating microbes, thus, it is likely that the viability of feed microbes in this study was higher than the previous studies [30, 86]. This may be responsible for the higher microbial overlap between the feeds and the intestine in the current study compared with earlier studies. However, it is reported that the feed processing (pre-conditioning *vs*. non-preconditioning) slightly influenced the gut microbiome of rainbow trout [91]. Further investigation on the impact of extrusion treatment on intestinal microbiota of fish fed yeast-based diets in Atlantic salmon may be needed in the future. In accordance with previous studies [25, 30], water had a lower impact in shaping the intestinal microbiota of fish than the feeds. Microbial overlap between water and the intestine in the current study was higher than reported for Atlantic salmon reared in freshwater [25, 30, 86]. In seawater, Atlantic salmon maintain osmoregulation by ingesting water to compensate for water loss to the hyperosmotic environment [92]. Water drinking ability of salmon reared in seawater may facilitate uptake of microbes from the rearing water, and thus, may be responsible for the higher microbial overlap between water and the intestine compared with previous studies in freshwater phase [25, 30, 86].

### Metabolic capacity of gut microbiota

The gut microbiota plays a critical role in host physiology by supporting growth performance, nutrient digestion, metabolism and participating in immune system maturation and pathogen defense [93, 94]. In the current study, a metagenome prediction tool was used to investigate the metabolic capacity of the gut microbiota of fish fed the experimental diets. The results revealed that the gut microbiota of fish fed ACJ diet were enriched in pathways related to mucin O-glycan degradation compared with fish fed the other diets. The gut microbiota of fish fed ACJ was dominated by *Pediococcus*, which has capability to adhere to intestinal mucus [95] and intestinal epithelial cells [96]. The breakdown of mucin glycans by the gut microbiota generates a pool of microbial products that can be beneficial for host mucus production and for immune and metabolic responses [21, 22]. This plays an important function in mucosal health, which is considered the first line of defense protecting the epithelial layer from pathogen invasion and other luminal compounds [21]. Our results further showed that pathways related to valerate metabolism were enriched in fish fed FM diet compared with fish fed ICJ, IWA and SBM diets. Valerate is a scarcely studied short chain fatty acid that can be produced as an end product of microbial fermentation [97]. The production of short chain fatty acids can act as link between the microbiota and the immune system by modulating the different aspects of intestinal epithelial cell [97, 98]. It has been reported that valerate production can help to inhibit the growth of *Clostridioides difficile,* both in vitro and in vivo [99], a bacterium that has been implicated in the development of inflammatory bowel disease in humans [100]. Although the role of valerate on fish physiology is not reported in literature, it is possible that increased valerate metabolism may be responsible for the normal intestinal health observed in fish fed FM diet in the current study [29].

Prediction tools are used to infer metabolic functions of gut microbiota produced through amplicon sequencing [101–103], but their validity is often questionable [103]. The GSMMs used in the current study were based on human gut microbiota, and the predicted metabolic capacities may not exactly mimic that of fish gut microbiota. Additionally, only about half of the identified ASVs were matched to a known GSMM, thus limiting the ability of the analysis to represent the whole gut microbiota of fish used in the present study. Based on these shortcomings, the results of the predicted metabolic capacity of fish gut microbiota reported in this study should be interpreted with caution.

## Conclusions

The present study showed that fifteen core microbial groups were detected in all dietary groups. The results showed that the richness and diversity of gut microbiota was lower in fish fed SBM compared with fish fed FM diet. The microbial composition and richness were similar among fish fed ICJ, IWA and SBM diets. Inclusion of autolyzed yeasts (ACJ and AWA) lowered the richness and diversity of gut microbiota in fish. Fish fed ACJ diet increased relative abundance of *Pediococcus*, and mucin O-glycan degradation pathway while fish fed AWA diet increased relative abundance of *Bacillaceae* compared with other diets. The results also suggest that the ameliorating effects of yeasts on SBMIE is related to their capability to stimulate immune cells rather than through modulation of intestinal microbiota in Atlantic salmon. Future research should focus on increasing our understanding of functional role of microbiota enhanced through inclusion of yeasts in fish diets.

## Supplementary Information

**Additional file 1. Fig. S1**. Rarefaction curves showing subsampling of sample into minimum sample sequence (1,604 sequence per sample). The rarefied amplicon sequence variants table was used for computation of Jaccard and unweighted Unifrac beta-diversity distances. FM – fishmeal-based; SBM – soybean meal-based; 4 experimental diets containing 300 g/kg SBM and 100 g/kg of ICJ – inactivated Cyberlindnera jadinii; ACJ – autolyzed C. jadinii; IWA – inactivated Wickerhamomyces anomalus; AWA – autolyzed W. anomalus diets. **Fig. S2**. Principal component (PC) analysis on standardized amplicon sequence variants (ASVs). Score plots for PC1 and PC2 (A) and PC1 and PC3 (B), mean scores with 95% confidence intervals for PC1 (C), PC2 (D), and PC3 (E), and percentage of variance explained by PCs (F). FM – fishmeal-based; SBM – soybean meal-based; 4 experimental diets containing 300 g/kg SBM and 100 g/kg of ICJ – inactivated Cyberlindnera jadinii; ACJ – autolyzed C. jadinii; IWA – inactivated Wickerhamomyces anomalus; AWA – autolyzed W. anomalus diets. **Fig. S3**. Principal component (PC) analysis on metabolic reaction abundances (z-scores). Score plots for PC1 and PC2 (A) and PC1 and PC3 (B), mean scores with 95% confidence intervals for PC1 (C), PC2 (D), and PC3 (E), and percentage of variance explained by PCs (F). FM – fishmeal-based; SBM – soybean meal-based; 4 experimental diets containing 300 g/kg SBM and 100 g/kg of ICJ – inactivated Cyberlindnera jadinii; ACJ – autolyzed C. jadinii; IWA – inactivated Wickerhamomyces anomalus; AWA – autolyzed W. anomalus diets. **Fig. S4**. Expected (mock_exp) and observed (mock and mock_1) taxonomic profiles of the mock microbial community standard. **Fig. S5**. Microbiota composition of water samples. Relative abundance of the top 10 most abundant taxa at phylum level (A) and top 15 most abundant taxa at genus or lowest taxonomic rank (B). The mean relative abundance of each taxon within the same water type is displayed on the right side. The samples are group water type; SW – water collected from the source tank (i.e., header tank) and TW – water collected from the fish rearing tanks. Water collected from the 18 fish tanks were mixed, four subsamples were taken and used for the microbiota analysis. **Fig. S6**. Venn’s diagram (A and B) showing the shared and the unique amplicon sequence variants (ASVs) in the digesta sample of fish fed the experimental diets. The ASVs were computed using a prevalence threshold of 80%. FM – fishmeal-based; SBM – soybean meal-based; 4 experimental diets containing 300 g/kg SBM and 100 g/kg of ICJ – inactivated Cyberlindnera jadinii; ACJ – autolyzed C. jadinii; IWA – inactivated Wickerhamomyces anomalus; AWA – autolyzed W. anomalus diets. **Fig. S7**. Boxplots for homogeneity of multivariate dispersions (PERMDISP) in gut microbiota of fish fed experimental diets. The PERMDISP test was based on; (A) Jaccard distance, (B) Unweighted Unifrac distance (C) Aitchison distance and (D) PhILR transformed Euclidean distance. The samples are grouped by diets; FM – fishmeal-based; SBM – soybean meal-based; 4 experimental diets containing 300 g/kg SBM and 100 g/kg of ICJ – inactivated Cyberlindnera jadinii; ACJ – autolyzed C. jadinii; IWA – inactivated Wickerhamomyces anomalus; AWA – autolyzed W. anomalus diets. **Fig. S8**. Number of ASVs mapped to genome-scale metabolic models. Number of samples matched to models at different taxonomic levels (a) and the number of models mapped to each sample by taxonomic level (b). **Fig. S9**. The t-statistic tests comparing reaction abundances between each pair of diets. The t-statistic for each reaction is shown along with the mean across all reactions with 95% confidence interval for all significantly enriched subsystems. FM – fishmeal-based; SBM – soybean meal-based; 4 experimental diets containing 300 g/kg SBM and 100 g/kg of ICJ – inactivated Cyberlindnera jadinii; ACJ – autolyzed C. jadinii; IWA – inactivated Wickerhamomyces anomalus; AWA – autolyzed W. anomalus diets. **Table S1**. Composition of spray-dried yeasts with and without the autolysis treatment. All values are presented in % DM, except gross energy which is presented in MJ/kg DM. **Table S4**. Pair-wise comparisons alpha-diversity indices of gut microbiota in Atlantic salmon smolts fed FM-based diet or SBM-based diet with yeasts. Table S5. PERMANOVA analysis for beta-diversity of gut microbiota in Atlantic salmon smolts fed FM-based diet or SBM-based diet with yeasts. Table S6. Test of homogeneity of multivariate dispersions among dietary groups.

**Additional file 2. Table S2**. The dominant taxa identified as contaminants in the negative controls and the blank filter papers. **Table S3**. The prevalence of core ASVs in the digesta of fish fed the experimental diets.

## Data Availability

The raw 16S rRNA gene sequence data and metadata files are deposited at the NCBI SRA database under the BioProject PRJNA797563. Other data and the code for reproducing the results are available in the Github repository https://github.com/Jeleel2020/Salmon_Yeasts_Microbiota.

## Abbreviations

ASVs: Amplicon sequence variants
CJ: *Cyberlindnera jadinii*
ICJ: Inactivated CJ
ACJ: Autolyzed CJ
WA: *Wickerhamomyces anomalus*
IWA: Inactivated WA
AWA: Autolyzed WA
BR: Broad range
FM: Fishmeal
GSMMs: Genome-scale metabolic models
HS: High sensitivity
LAB: Lactic acid bacteria
MOS: Mannan oligosaccharides
PCA: Principal component analysis
PCoA: Principal coordinates analysis
PD: Phylogenetic diversity
PERMANOVA: Permutation multivariate analysis of variance
SBM: Soybean meal
SBMIE: SBM-induced enteritis
SPC: Soy protein concentrates
