# Supplementary material for: Effect of yeast species and processing on intestinal microbiota of Atlantic salmon (Salmo salar) fed soybean meal-based diets in seawater
Source: Anim Microbiome. 2023 Apr 4;5:21. doi: 10.1186/s42523-023-00242-y (PMC10074822; doi:10.1186/s42523-023-00242-y)
Supplement: Supplementary file 1 — Additional file 1. Fig. S1. Rarefaction curves showing subsampling of sample into minimum sample sequence (1,604 sequence per sample). The rarefied amplicon sequence variants table was used for computation of Jaccard and unweighted Unifrac beta-diversity distances. FM – fishmeal-based; SBM – soybean meal-based; 4 experimental diets containing 300 g/kg SBM and 100 g/kg of ICJ – inactivated Cyberlindnera jadinii; ACJ – autolyzed C. jadinii; IWA – inactivated Wickerhamomyces anomalus; AWA – autolyzed W. anomalus diets. Fig. S2. Principal component (PC) analysis on standardized amplicon sequence variants (ASVs). Score plots for PC1 and PC2 (A) and PC1 and PC3 (B), mean scores with 95% confidence intervals for PC1 (C), PC2 (D), and PC3 (E), and percentage of variance explained by PCs (F). FM – fishmeal-based; SBM – soybean meal-based; 4 experimental diets containing 300 g/kg SBM and 100 g/kg of ICJ – inactivated Cyberlindnera jadinii; ACJ – autolyzed C. jadinii; IWA – inactivated Wickerhamomyces anomalus; AWA – autolyzed W. anomalus diets. Fig. S3. Principal component (PC) analysis on metabolic reaction abundances (z-scores). Score plots for PC1 and PC2 (A) and PC1 and PC3 (B), mean scores with 95% confidence intervals for PC1 (C), PC2 (D), and PC3 (E), and percentage of variance explained by PCs (F). FM – fishmeal-based; SBM – soybean meal-based; 4 experimental diets containing 300 g/kg SBM and 100 g/kg of ICJ – inactivated Cyberlindnera jadinii; ACJ – autolyzed C. jadinii; IWA – inactivated Wickerhamomyces anomalus; AWA – autolyzed W. anomalus diets. Fig. S4. Expected (mock_exp) and observed (mock and mock_1) taxonomic profiles of the mock microbial community standard. Fig. S5. Microbiota composition of water samples. Relative abundance of the top 10 most abundant taxa at phylum level (A) and top 15 most abundant taxa at genus or lowest taxonomic rank (B). The mean relative abundance of each taxon within the same water type is displayed on the right side. The samples a [file 42523_2023_242_MOESM1_ESM.pdf]

Fig. S1.

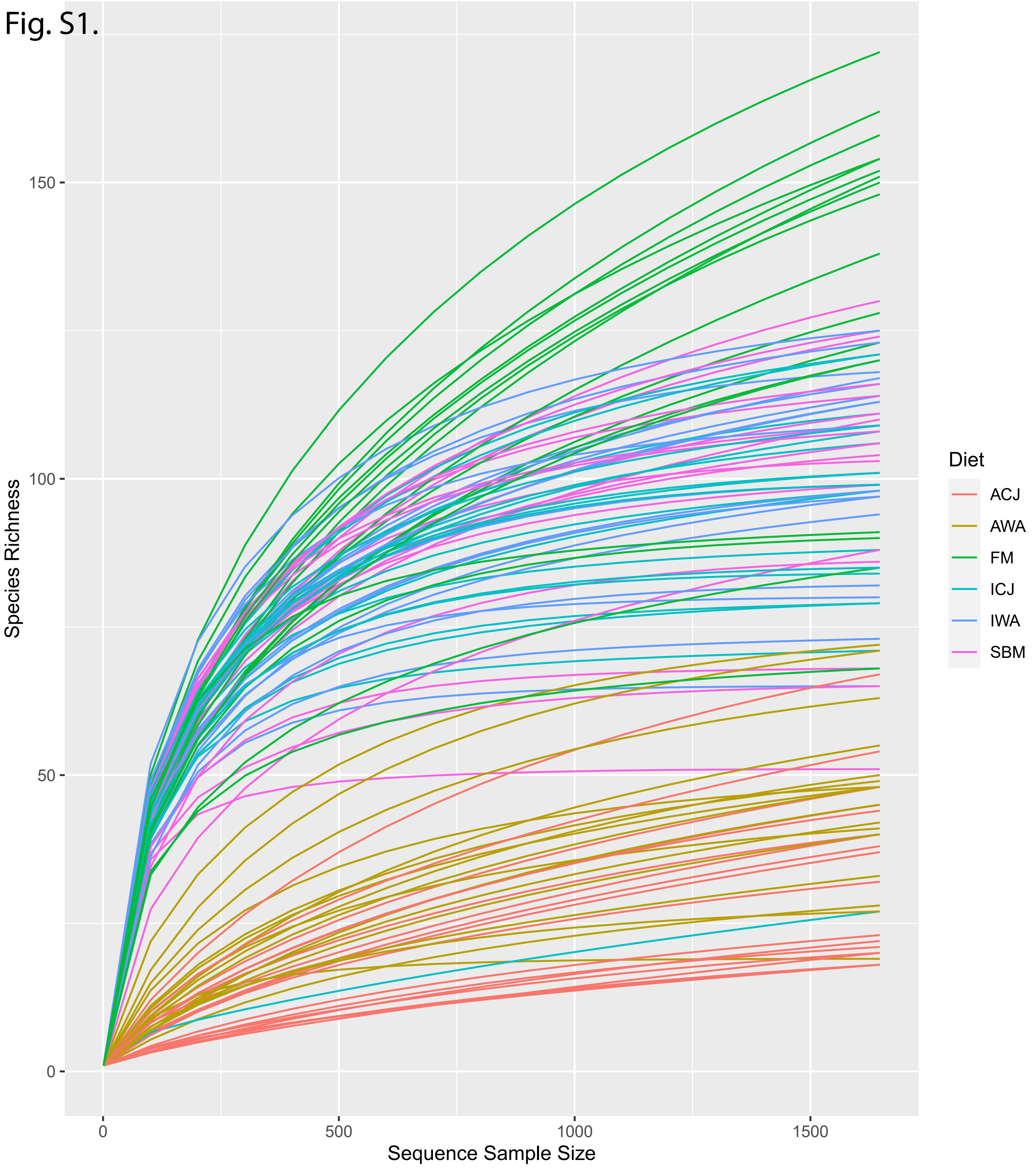

Species Richness
Sequence Sample Size
0
50
100
150
0
500
1000
1500
Diet
ACJ
AWA
FM
ICJ
IWA
SBM

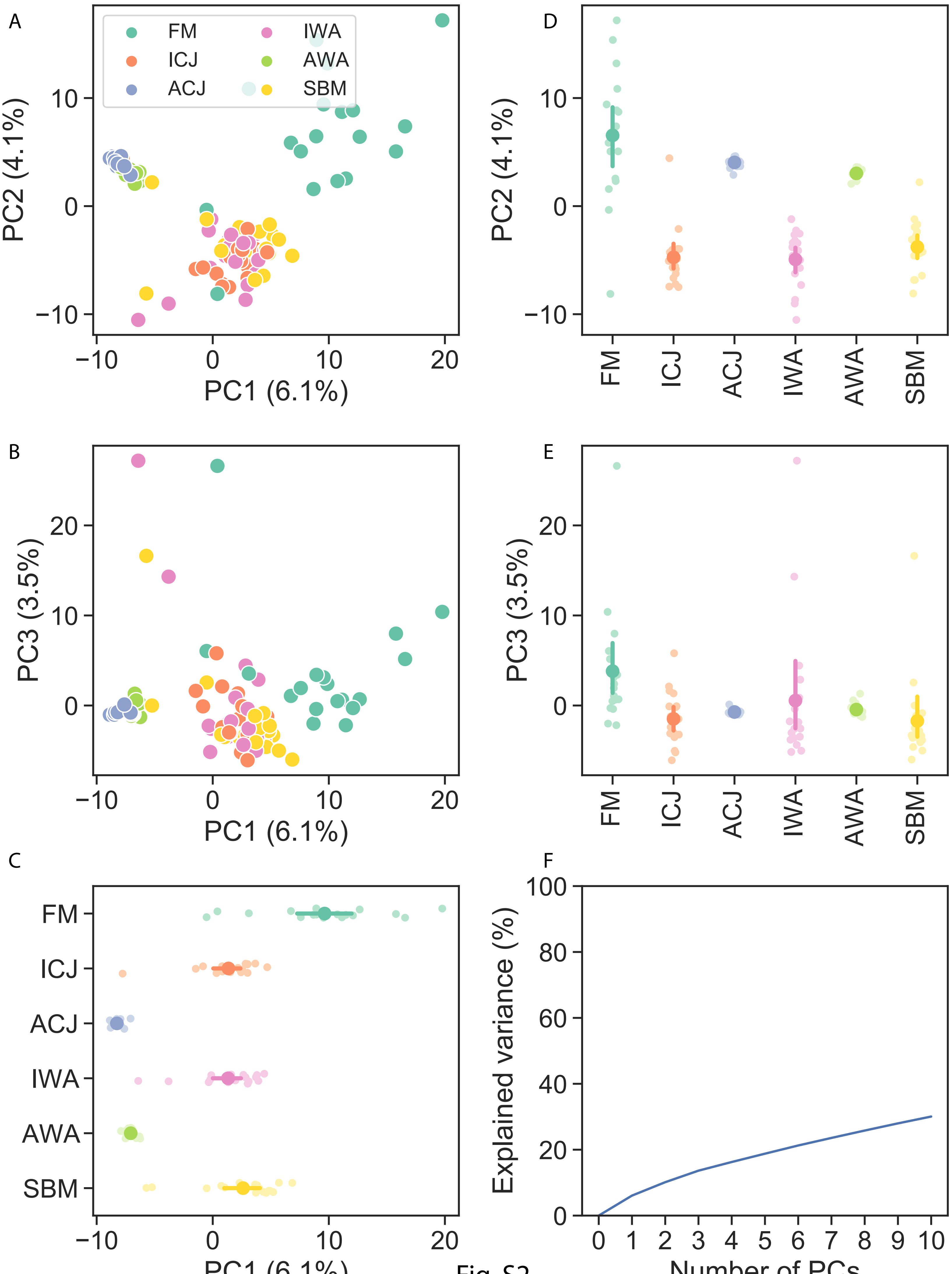
A
FM
ICJ
ACJ
IWA
AWA
SBM
PC2 (4.1%)
PC1 (6.1%)
B
PC3 (3.5%)
PC1 (6.1%)
C
FM
ICJ
ACJ
IWA
AWA
SBM
PC1 (6.1%)
D
PC2 (4.1%)
FM
ICJ
ACJ
IWA
AWA
SBM
E
PC3 (3.5%)
FM
ICJ
ACJ
IWA
AWA
SBM
F
Explained variance (%)
Number of PCs

Fig. S2.

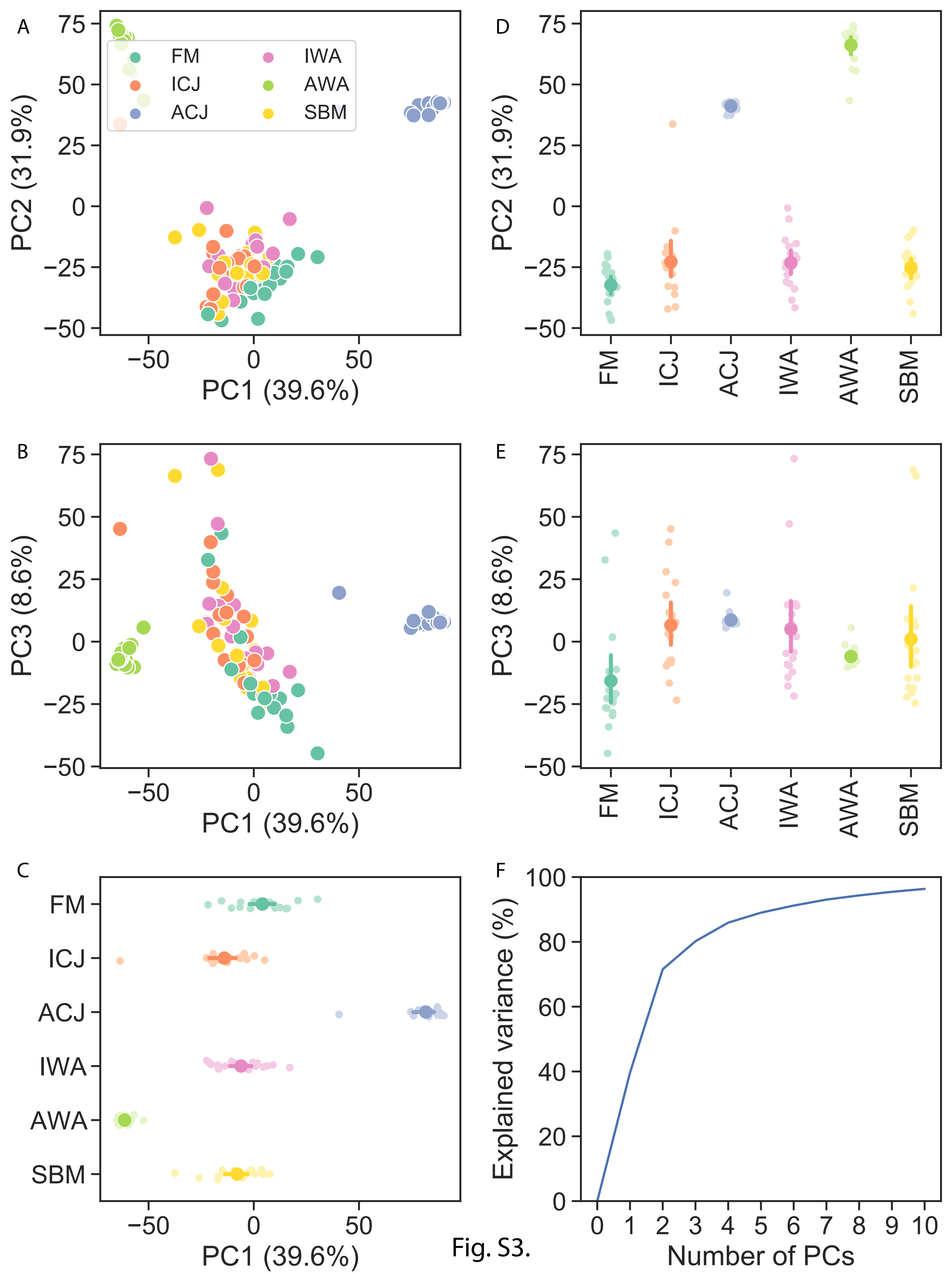

A
B
C
D
E
F
FM
ICJ
ACJ
IWA
AWA
SBM
PC1 (39.6%)
PC2 (31.9%)
PC3 (8.6%)
Explained variance (%)
Number of PCs

Fig. S3.

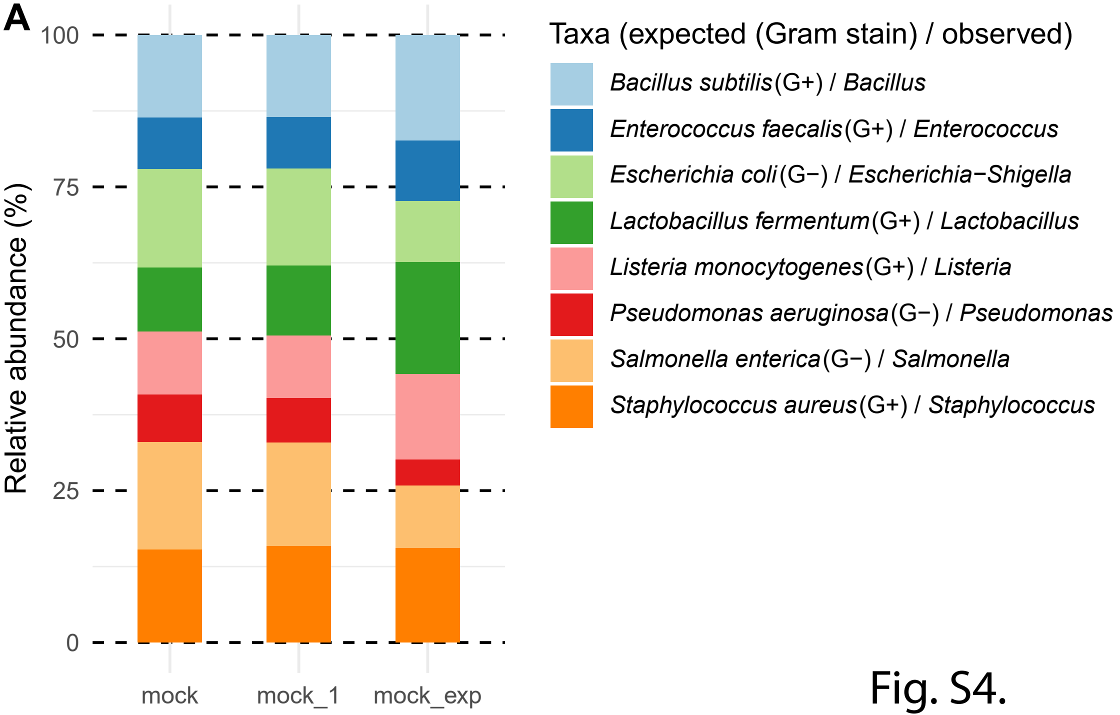
A
Relative abundance (%)
100
75
50
25
0
mock
mock_1
mock_exp
Taxa (expected (Gram stain) / observed)
Bacillus subtilis(G+) / Bacillus
Enterococcus faecalis(G+) / Enterococcus
Escherichia coli(G−) / Escherichia−Shigella
Lactobacillus fermentum(G+) / Lactobacillus
Listeria monocytogenes(G+) / Listeria
Pseudomonas aeruginosa(G−) / Pseudomonas
Salmonella enterica(G−) / Salmonella
Staphylococcus aureus(G+) / Staphylococcus

Fig. S4.

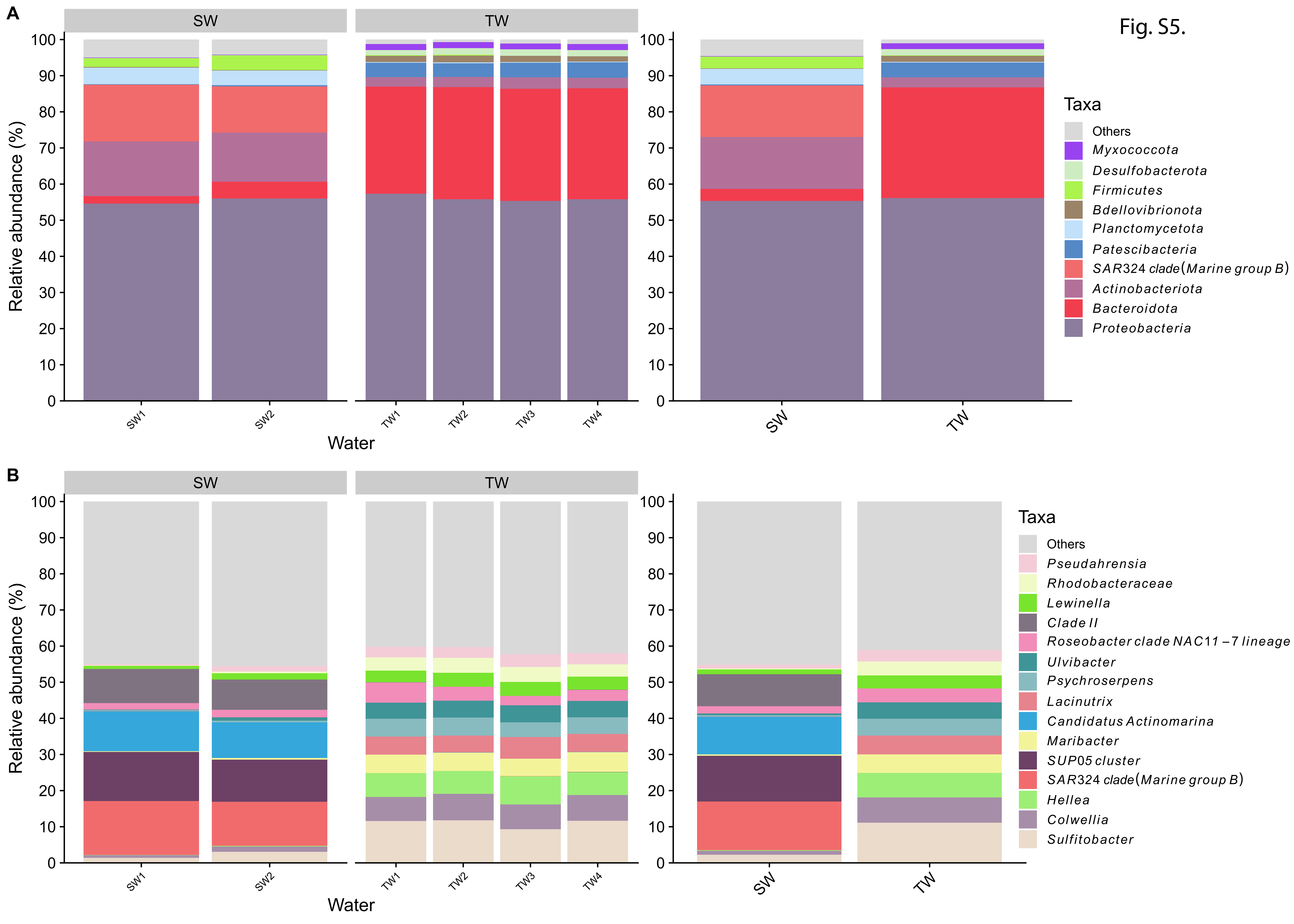
Fig. S5.
A
B
SW
TW
Relative abundance (%)
0
10
20
30
40
50
60
70
80
90
100
SW1
SW2
TW1
TW2
TW3
TW4
Water
SW
TW
Taxa
Others
Myxococcota
Desulfobacterota
Firmicutes
Bdellovibrionota
Planctomycetota
Patescibacteria
SAR324 clade(Marine group B)
Actinobacteriota
Bacteroidota
Proteobacteria
Taxa
Others
Pseudahrensia
Rhodobacteraceae
Lewinella
Clade II
Roseobacter clade NAC11−7 lineage
Ulvibacter
Psychroserpens
Lacinutrix
Candidatus Actinomarina
Maribacter
SUP05 cluster
SAR324 clade(Marine group B)
Hellea
Colwellia
Sulfitobacter

Fig. S6.

A

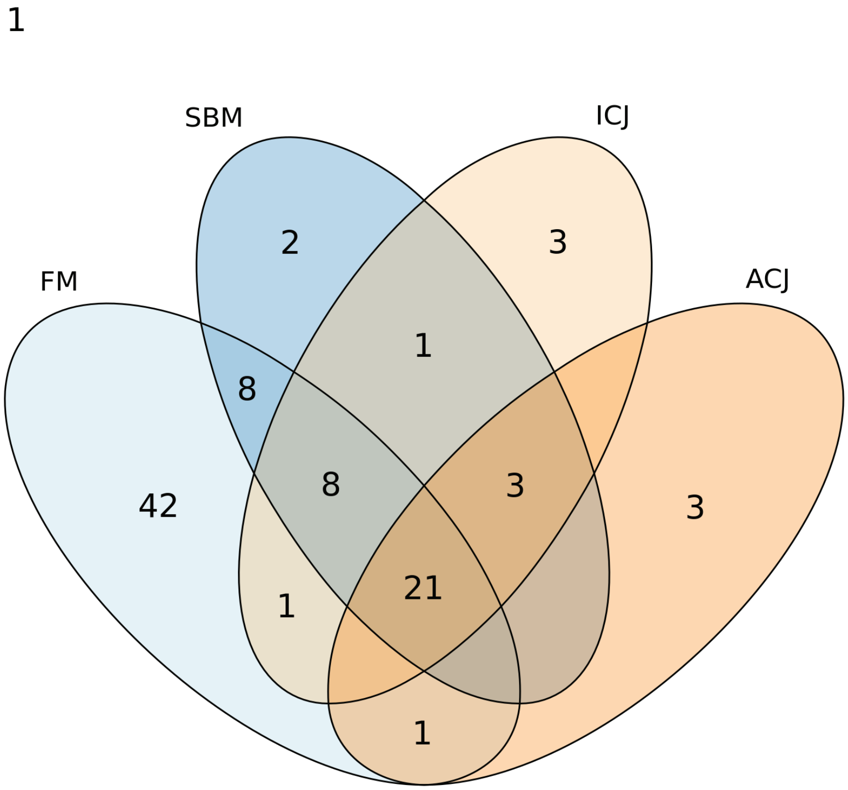
1
SBM
ICJ
FM
ACJ
2
3
1
8
8
3
42
3
21
1
1

B

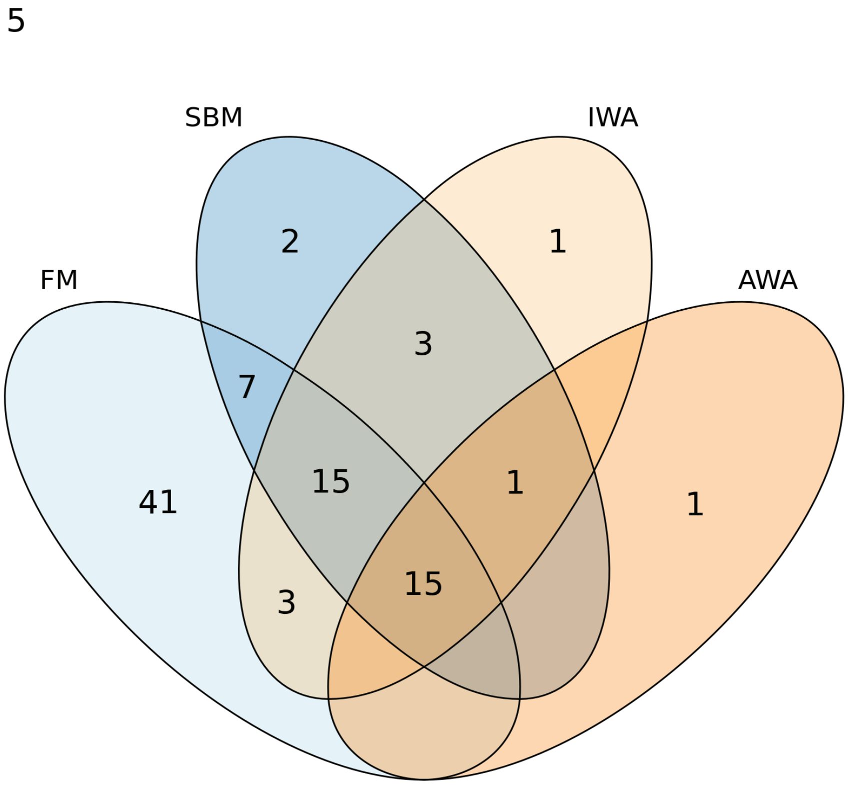
5
SBM
IWA
FM
AWA
2
1
3
7
15
1
41
1
15
3

Fig. S7.

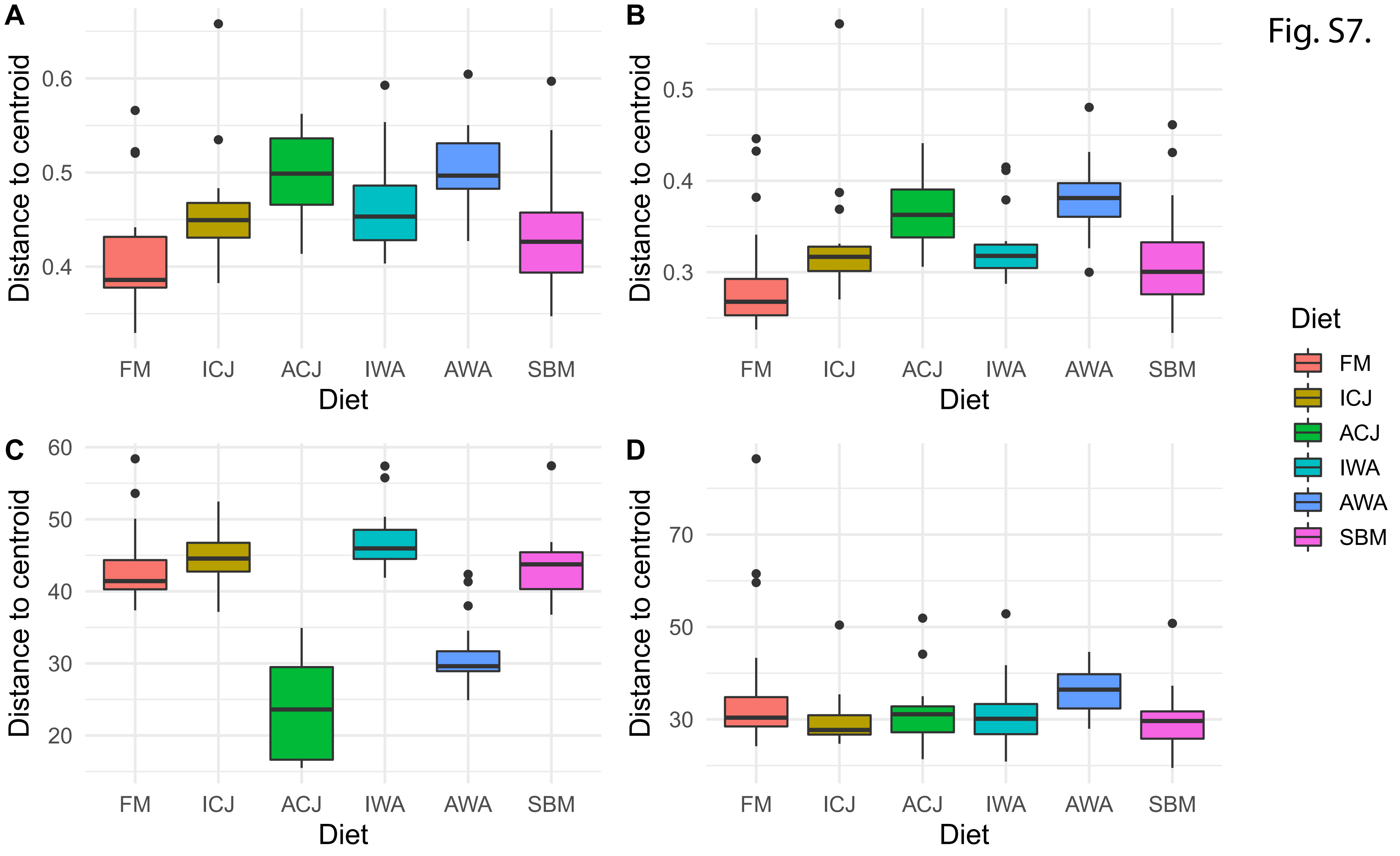

A
B
C
D
Distance to centroid
0.4
0.5
0.6
0.3
0.4
0.5
20
30
40
50
60
30
50
70
FM
ICJ
ACJ
IWA
AWA
SBM
Diet
Diet
FM
ICJ
ACJ
IWA
AWA
SBM

Fig. S8.

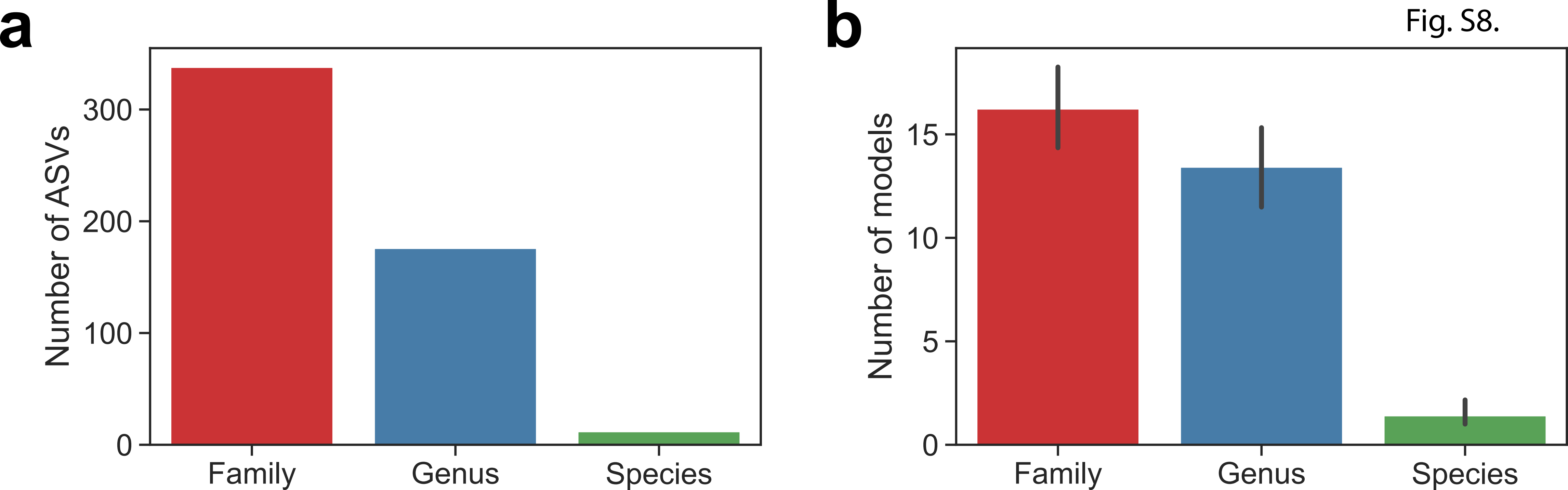
a
b
Number of ASVs
0
100
200
300
Family
Genus
Species
Number of models
0
5
10
15
Family
Genus
Species

Fig. S9.

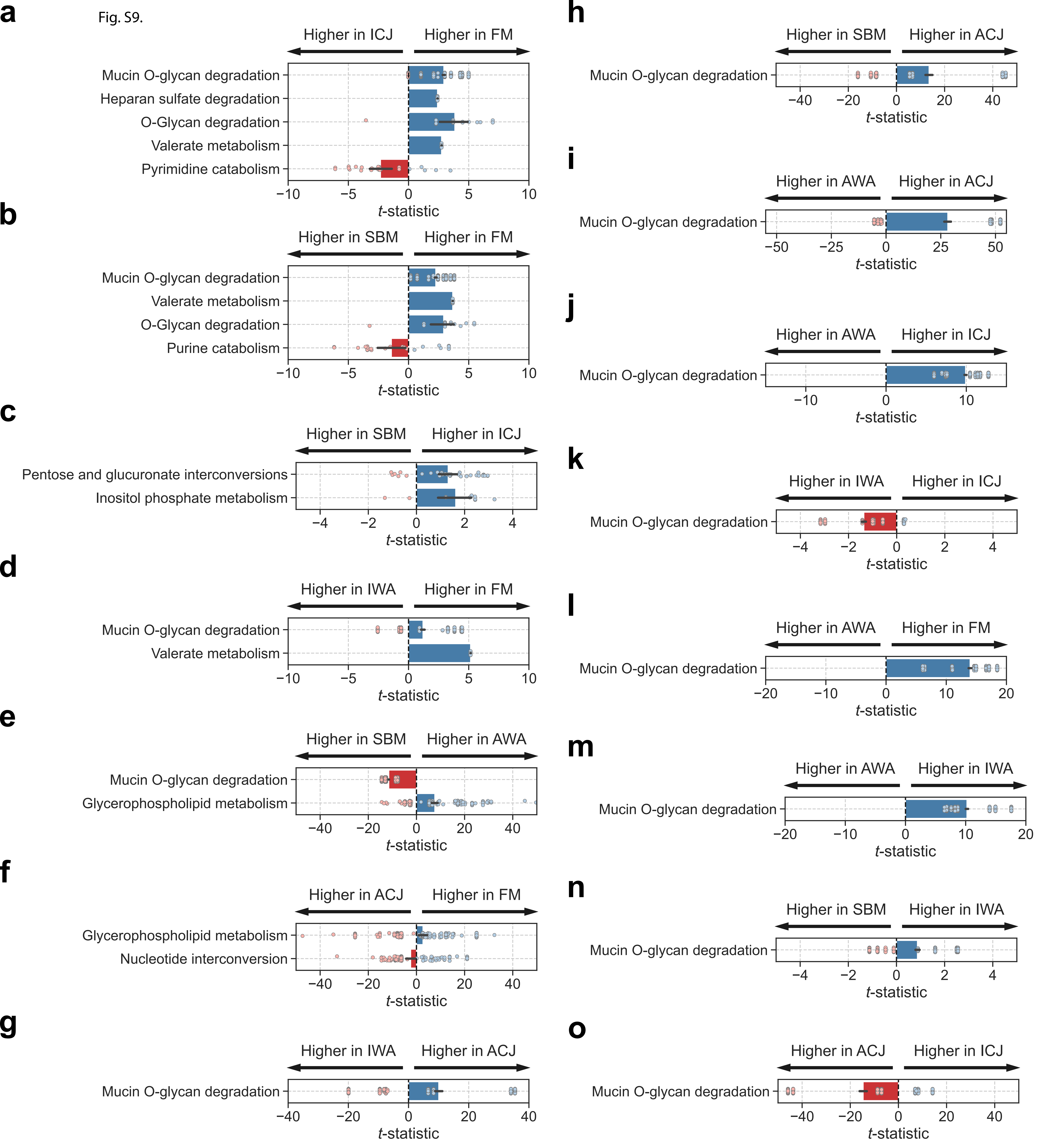
a
Higher in ICJ
Higher in FM
Mucin O-glycan degradation
Heparan sulfate degradation
O-Glycan degradation
Valerate metabolism
Pyrimidine catabolism
t-statistic
b
Higher in SBM
Higher in FM
Mucin O-glycan degradation
Valerate metabolism
O-Glycan degradation
Purine catabolism
t-statistic
c
Higher in SBM
Higher in ICJ
Pentose and glucuronate interconversions
Inositol phosphate metabolism
t-statistic
d
Higher in IWA
Higher in FM
Mucin O-glycan degradation
Valerate metabolism
t-statistic
e
Higher in SBM
Higher in AWA
Mucin O-glycan degradation
Glycerophospholipid metabolism
t-statistic
f
Higher in ACJ
Higher in FM
Glycerophospholipid metabolism
Nucleotide interconversion
t-statistic
g
Higher in IWA
Higher in ACJ
Mucin O-glycan degradation
t-statistic
h
Higher in SBM
Higher in ACJ
Mucin O-glycan degradation
t-statistic
i
Higher in AWA
Higher in ACJ
Mucin O-glycan degradation
t-statistic
j
Higher in AWA
Higher in ICJ
Mucin O-glycan degradation
t-statistic
k
Higher in IWA
Higher in ICJ
Mucin O-glycan degradation
t-statistic
l
Higher in AWA
Higher in FM
Mucin O-glycan degradation
t-statistic
m
Higher in AWA
Higher in IWA
Mucin O-glycan degradation
t-statistic
n
Higher in SBM
Higher in IWA
Mucin O-glycan degradation
t-statistic
o
Higher in ACJ
Higher in ICJ
Mucin O-glycan degradation
t-statistic

Table S1. Composition of spray-dried yeasts with and without the autolysis treatment. All values are presented in % DM, except gross energy which is presented in MJ/kg DM.

	Cyberlindnera jadinii		*Wickerhamomyces anomalus*	
	Inactivated	Autolyzed	Inactivated	Autolyzed
DM[1] (%)	96.3 ± 0.03	93.1 ± 0.04	96.1 ± 0.02	96.1 ± 0.06
Nutrients (% DM)[2]				
Crude protein	46.5 ± 0.47	47.4 ± 0.01	43.0 ± 0.04	42.1 ± 0.26
Crude lipids	2.9 ± 0.18	5.7 ± 0.17	2.8 ± 0.06	4.1 ± 0.02
Ash	5.7 ± 0.00	5.9 ± 0.01	5.5 ± 0.00	5.5 ± 0.00
Total phosphorus	0.6 ± 0.02	0.6 ± 0.01	0.5 ± 0.01	0.4 ± 0.02
Gross energy (MJ/kg DM)	21.8 ± 0.01	22.32 ± 0.02	21.1 ± 0.01	21.5 ± 0.01
Cell wall polysaccharides (% DM)[3]				
β-glucan	16.4 ± 3.19	11.1 ± 0.84	15 ± 1.41	11.8 ± 0.73
Mannan	7.9 ± 2.16	6.0 ± 0.66	11.3 ± 0.95	10.4 ± 0.67
Chitin	0.3 ± 0.07	0.2 ± 0.02	0.5 ± 0.05	0.4 ± 0.08

[1]DM – dry matter.
[2]Crude protein, crude lipids, ash, total phosphorus, and gross energy contents of yeasts are mean values ± SD from duplicate analyses.
[3]β-glucan, mannan and chitin contents of yeasts are mean values ± SD from triplicate analyses.

Table S4. Pair-wise comparisons of alpha-diversity indices of gut microbiota in Atlantic salmon smolts fed FM-based diet or SBM-based diet with yeasts.[1]

	Observed ASVs	Pielou's evenness	Shannon's index	Faith's PD
P-values[2]	< 0.0001	< 0.0001	< 0.0001	< 0.0001
Pair-wise comparisons[3]				
IWAvsAWA	< 0.0001	< 0.0001	< 0.0001	< 0.0001
IWAvsACJ	0.003	< 0.0001	< 0.0001	0.001
IWAvsSBM	0.81	1.000	0.970	0.740
IWAvsICJ	0.77	1.000	0.970	0.740
IWAvsFM	0.001	1.000	< 0.0001	< 0.0001
AWAvsACJ	0.62	0.052	0.007	0.580
AWAvsSBM	< 0.0001	< 0.0001	< 0.0001	< 0.0001
AWAvsICJ	< 0.0001	< 0.0001	< 0.0001	< 0.0001
AWAvsFM	< 0.0001	< 0.0001	< 0.0001	< 0.0001
ACJvsSBM	0.010	< 0.0001	< 0.0001	0.003
ACJvsICJ	0.010	< 0.0001	< 0.0001	0.002
ACJvsFM	< 0.0001	< 0.0001	< 0.0001	< 0.0001
SBMvsICJ	0.620	1.000	0.970	0.500
SBMvsFM	0.002	1.000	0.001	0.001
ICJvsFM	< 0.0001	0.910	< 0.0001	< 0.0001

[1]The diets are: FM – fishmeal-based; SBM – soybean meal-based; 4 other diets containing 300 g/kg SBM and 100 g/kg of ICJ – inactivated *Cyberlindnera jadinii*; ACJ – autolyzed *C. jadinii*; IWA – inactivated *Wickerhamomyces anomalus*; AWA – autolyzed *W. anomalus* diets.

[2] P-values computed for diet effect with Kruska-walis test.

[3] Wilcox pairwise comparison to identify differences between diets.

Table S5. PERMANOVA analysis for beta-diversity of gut microbiota in Atlantic salmon smolts fed FM-based diet or SBM-based diet with yeasts.[1]

	Jaccard distance[2]	Unweighted UniFrac distance[2]	Robust aitchison distance[3]	Phylogenetic isometric log-ratio (PHILR) transformed Euclidean distance[3]
P-values[3]	< 0.001	< 0.001	< 0.001	< 0.001
Pair-wise comparisons[4]				
IWAvsAWA	0.015	0.015	0.015	0.015
IWAvsACJ	0.015	0.015	0.015	0.015
IWAvsSBM	0.600	0.27	0.075	0.015
IWAvsICJ	1.000	1.000	1.000	0.345
IWAvsFM	0.015	0.015	0.015	0.015
AWAvsACJ	0.015	0.015	0.015	0.015
AWAvsSBM	0.015	0.015	0.015	0.015
AWAvsICJ	0.015	0.015	0.015	0.015
AWAvsFM	0.015	0.015	0.015	0.015
ACJvsSBM	0.015	0.015	0.015	0.015
ACJvsICJ	0.015	0.015	0.015	0.015
ACJvsFM	0.015	0.015	0.015	0.015
SBMvsICJ	0.600	1.000	0.195	1.000
SBMvsFM	0.015	0.015	0.015	0.015
ICJvsFM	0.015	0.015	0.015	0.015

[1]The diets are: FM – fishmeal-based; SBM – soybean meal-based; 4 other diets containing 300 g/kg SBM and 100 g/kg of ICJ – inactivated *Cyberlindnera jadinii*; ACJ – autolyzed *C. jadinii*; IWA – inactivated *Wickerhamomyces anomalus*; AWA – autolyzed *W. anomalus* diets.

[2] Performed on phyloseq object rarefied to minimum read sequence in the sample.

[3] Performed on unrarefied phyloseq object.

[4] P-values of permutational multivariate analysis of variance (PERMANOVA) test for the four beta-diversity distances.

[3] PERMANOVA pairwise comparisons for the four beta-diversity distances.

Table S6. Test of homogeneity of multivariate dispersions among dietary groups.

	Jaccard distance[2]	Unweighted UniFrac distance[2]	Robust aitchison distance[3]	Phylogenetic isometric log-ratio (PHILR) transformed Euclidean distance[3]
P-values[4]	0.001	0.001	0.001	0.002
Pair-wise comparisons[5]				
IWAvsAWA	<0.001	0.023	<0.001	0.018
IWAvsACJ	<0.001	0.032	<0.001	0.824
IWAvsSBM	0.412	0.195	0.039	0.668
IWAvsICJ	0.84	0.9	0.109	0.648
IWAvsFM	0.051	0.009	0.041	0.150
AWAvsACJ	0.328	0.8	0.017	0.028
AWAvsSBM	0.001	0.002	0.001	0.002
AWAvsICJ	0.001	0.026	0.001	0.001
AWAvsFM	0.001	0.001	0.001	0.857
ACJvsSBM	0.007	0.002	0.001	0.522
ACJvsICJ	0.058	0.041	0.001	0.508
ACJvsFM	0.001	0.001	0.001	0.205
SBMvsICJ	0.418	0.24	0.471	0.950
SBMvsFM	0.333	0.214	0.842	0.085
ICJvsFM	0.086	0.013	0.402	0.083

[1]The diets are: FM – fishmeal-based; SBM – soybean meal-based; 4 other diets containing 300 g/kg SBM and 100 g/kg of ICJ – inactivated *Cyberlindnera jadinii*; ACJ – autolyzed *C. jadinii*; IWA – inactivated *Wickerhamomyces anomalus*; AWA – autolyzed *W. anomalus* diets.

[2] Performed on phyloseq object rarefied to minimum read sequence in the sample.

[3] Performed on unrarefied phyloseq object.

[4] P-values of homogeneity of multivariate dispersions using PERMDISP test for the four beta-diversity distances.

[5] PERMDISP pairwise comparisons for the four beta-diversity distances.
